# 17β-estradiol alleviated ferroptotic neuroinflammation by suppressing ATF4 in mouse model of Parkinson’s disease

**DOI:** 10.1038/s41420-024-02273-z

**Published:** 2024-12-19

**Authors:** Guoming Wang, Wenxin Zhuang, Yijun Zhou, Xu Wang, Zhenfeng Li, Chuanliang Liu, Wentong Li, Maotao He, E. Lv

**Affiliations:** 1 School of Clinical Medicine, Shandong Second Medical University, Weifang, Shandong Province China; 2 Experimental Center for Medical Research, Shandong Second Medical University, Weifang, Shandong Province China; 3 College of Computer Science and Engineering, Shandong University of Science and Technology, Qingdao, Shandong Province China; 4 Department of Geriatrics, Weifang People’s Hospital, Weifang, China; 5 Department of Pathology, School of Basic Medicine Sciences, Shandong Second Medical University, Weifang, Shandong Province China; 6 Department of Histoembryology, School of Basic Medicine Sciences, Shandong Second Medical University, Weifang, Shandong Province China

**Keywords:** Cell biology, Neuroimmunology

## Abstract

Neuroinflammation induced by activation of microglial is a vital contributor to progression of Parkinson’s disease (PD), emerging evidences suggested that ferroptosis played a pivotal role in microglial activation and subsequent dopaminergic neuron loss. Nevertheless, the fundamental pathogenesis of that ferroptosis contributes to PD is not yet sufficiently understood. Based on GEO dataset, ferroptosis related genes were found to be enriched in PD patients and MPTP mouse model of PD, among them, ATF4 was found to be dramatically differentially expressed. In our study, ectopic expression of ATF4 augmented MPP^+^-induced cytotoxic and activation of BV2 cells with upregulated intracellular L-ROS, TLR4 and pNF-κB. Ectopic ATF4 effectively promoted transformation of microglial into M1 pro-inflammatory phenotype. 17β-estradiol (E2) attenuated expression of ATF4 in BV2 cells, silence of ATF4 enhanced protective effect of E2 on MPP^+^-treated BV2 cells. In MPTP-induced PD mouse model, administration of E2 further abated expression of ATF4 and inhibited expressions of pro-inflammatory cytokines and activation of TLR4/NF-κB pathway. Overall, E2 effectively counteracted TLR4/NF-κB signaling pathway by restraining ATF4 and inhibited inflammatory response triggered by ferroptosis, ultimately exerted anti-PD effects.

## Introduction

Parkinson’s disease (PD) is a common and progressive neurodegenerative disorder and thought to be provoked by a combination of genetic and environmental factors [1]. Ferroptosis, a regulated iron dependent cell death involving lethal accumulations of excessive reactive oxygen species (ROS)-mediated lipid peroxides, shares several features with PD pathophysiology [2]. ROS promoted neuroinflammation by activating NF-κB signaling pathway which was closely related to exaggeration of neurodegenerative diseases by inducing pro-inflammatory cytokines.

Microglia-mediated neuroinflammation is a common feature of PD [3, 4], it was reported that microglia governed central nervous system immune defense in nearly all neurological diseases, microglia have been recognized as the most important source of ROS which underlies microglial functions, especially M1 polarization [5]. In lipopolysaccharide (LPS)-stimulated microglia, eukaryotic translation initiation factor 2α (eIF2α)-activating transcription factor 4 (ATF4) pathway played significant roles in production of IL-1β, IL-6, and TNF-α [6]. Microglia phenotype transition from anti-inflammatory to pro-inflammatory was observed in LPS-mediated cognitive and motor dysfunction in mice via TLR4/NF-κB signaling pathway [7, 8].

Estrogen improves antioxidant status via interaction with estrogen receptors (ERs) to regulate expressions of antioxidant genes. Increasing amount of evidence supported neuroprotective role of estrogens [9], estrogen was suggested to contribute to neuronal survival and regulate inflammatory responses of microglial and astroglial [10, 11]. Both membrane and nuclear ERs are involved in 17β-estradiol (E2) neuroprotective function via regulating ROS level [12].

Here, ferroptosis was found to be a major regulated cell death of microglia exposed to 1-methyl-4-phenylpyridinium (MPP^+^) stress based on Gene Expression Omnibus (GEO) dataset, ectopic expression of ATF4 was confirmed in MPP^+^ treated microglia and microglia of PD patients. Moreover, we verified anti-neuroinflammatory effects of E2 via modulating ATF4 and suppressing TLR4/NF-κB inflammatory pathway in MPTP mouse model of PD.

## Results

### ATF4 was identified as a momentous DEGs in PD by bioinformatics analysis

**Fig. 1:**
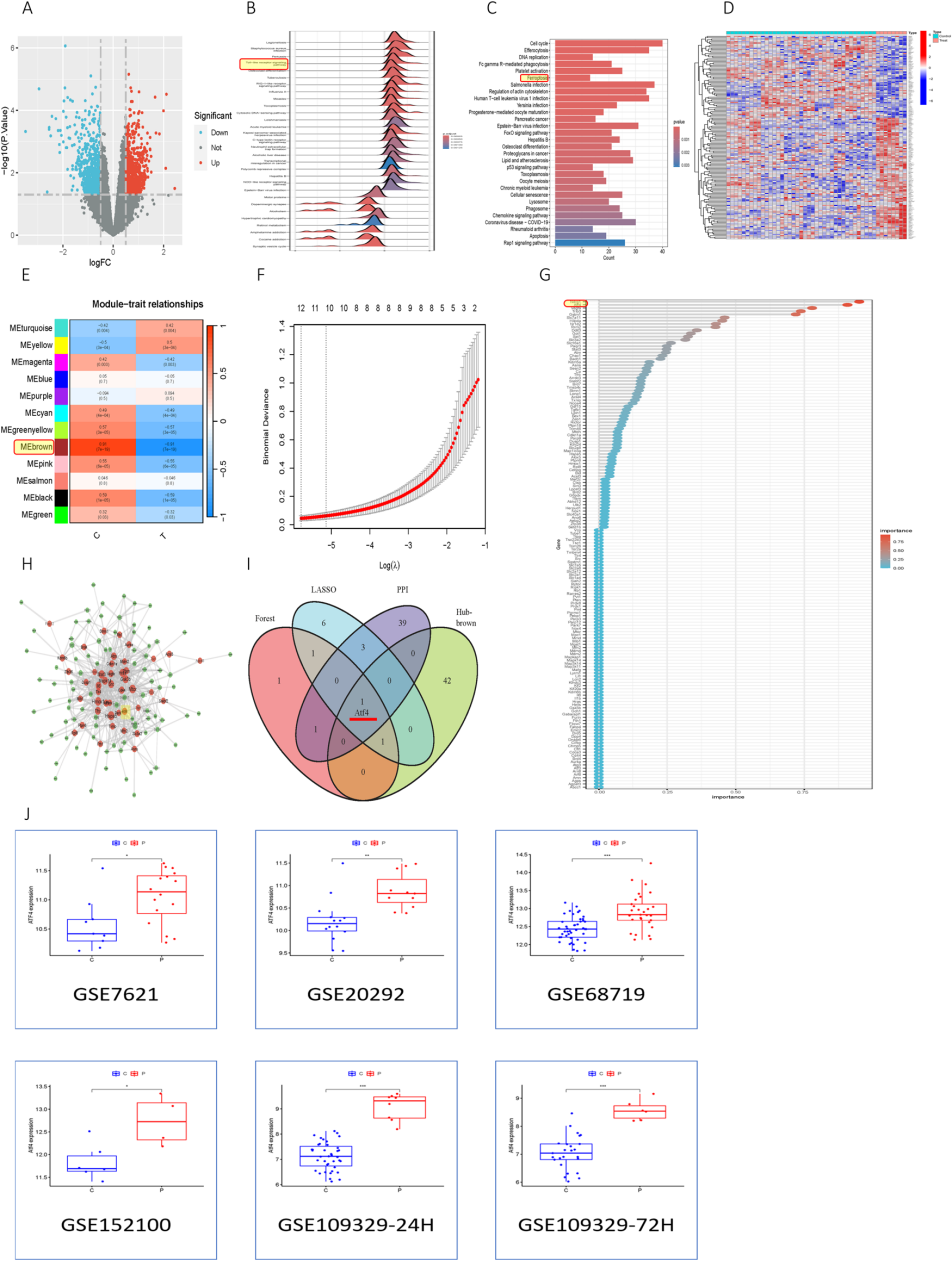
ATF4 was identified as a potential key gene in PD by bioinformatics. **A** Analysis volcano plot of DEGs from GSE7621. **B** Ridge plots showing the top enriched biological process of KEGG in GSE7621. **C** KEGG enrichment result of 1474 DEGs identified in GSE109329. **D** Heatmap of 154 ferroptosis-related DEGs in GSE109329. **E** Results of Module-trait relationships based on GSE109329 by WGCNA. **F** The most proper log value in LASSO model. **(G)** The RF module based on ferroptosis-related DEGs. **H** Results of PPI analysis. **I** Results of Venn diagram. **J** Expression profiles of ATF4 in GEO dataset.

To unravel the critical signaling pathways in PD, transcriptome microarray data of GSE7621 were retrieved and 1425 DEGs were screened, including 732 downregulated and 693 upregulated genes (Fig. 1A). Results of KEGG revealed a significant enrichment of inflammation and immunity pathways, specifically Toll-like receptor (TLR) and RIG-I-like receptor signaling pathways (Fig. 1B). Given the pivotal role of microglial cells in brain inflammation, we analyzed the GSE109329 dataset to explore changes in microglia in PD. After removing the significantly different G502 group and normalizing the data, we performed differential analysis. (Figure S1A-S1D), results of KEGG revealed significant enrichment of DEGs in ferroptosis pathway (Fig. 1C). We analyzed expressions of FRGs and constituted a new gene expression matrix using ‘limma’ R package (Fig. 1D). Subsequently, WGCNA-hub genes were collected using ‘WGCNA’ R package (Fig. 1E, Figure S1E-S1G), potential target genes were selected with ‘glmnet’ R package (Fig. 1F, Figure S1H), top 5 genes from 154 potential genes were identified using ‘random Forest’ R package according to RF algorithm (Fig. 1G), protein-protein interaction (PPI) analysis was performed with ‘STRINGdb’ R package (Fig. 1H, Figure S1I). Ultimately, by intersecting WGCNA-hub genes, lasso-selected genes, the top 5 genes and PPI-hub genes, ATF4 was discovered as a potential core key gene (Fig. 1I). Significant upregulation of ATF4 was confirmed in SN of PD patients and in vitro PD model by GSE20292, GSE68719, GSE152100 database (Fig. 1J).

### ATF4 augmented MPP^+^ induced ferroptosis of BV2 cells

**Fig. 2:**
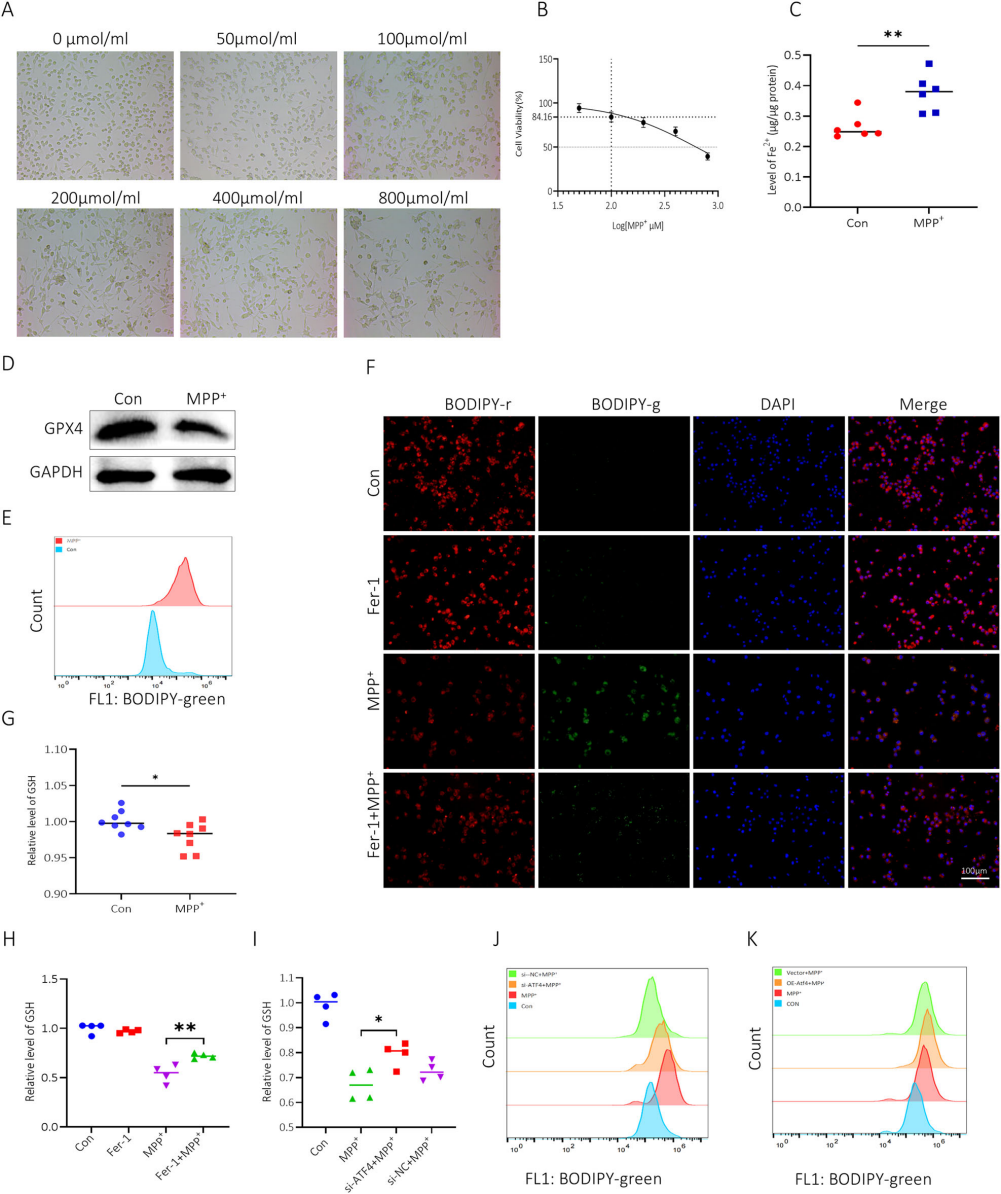
ATF4 accelerated MPP^+^-induced cytotoxic on BV2 cells. **A** Morphological changes of BV2 cells were observed under bright-field microscopy after 24 h of treatment with different concentrations of MPP^+^. **B** Cell viability of BV2 cells was assessed using the CCK-8 assay following gradient drug treatments. **C** Intracellular ferrous iron. **D** Expression of GPX4 by western blot in BV2 cells. **E**, **F** Levels of L-ROS in BV2 cells treated with MPP^+^ for 24 h, measured by FCM (**E**) and fluorescence microscopy (**F**). **G**–**I** Levels of GSH in BV2 cells under different treatments. **J**, **K** Levels of L-ROS in BV2 cells under different treatments. *P < 0.05, **P < 0.01.

Morphological changes of BV2 cells treated with MPP^+^ were observed, displaying cytotoxic effects characterized by a noticeable decrease in cell number, prolonged doubling time, enlargement in cell volume, irregular morphology, and increased thickness and number of cellular processes (Fig. 2A). At a concentration of 100 μM, MPP^+^ exhibited significant cytotoxic activity against BV2 cells, as evidenced by marked changes in cell morphology, yet cellular viability remained above 80% (Fig. 2B). Therefore, we maintained this concentration of MPP^+^ for subsequent experiments. We measured the effects of ferroptotic stimuli directly on microglia cells by MPP^+^, as shown in Fig. 2C, Fe^2+^ was considerably increased in BV2 cells treated with MPP^+^, whereas GPX4 was prominently declined in MPP^+^-treated cells (Fig. 2D). As shown in Fig. 2E, F and Figure S2A, production of L-ROS was increased by 601.4% in MPP^+^-treated BV2 cells. Meanwhile MPP^+^ decreased intracellular GSH (Fig. 2G). MPP^+^-induced ferroptotic stimuli was rescued by ferroptosis inhibitor Fer-1 (Fig. 2H). Then, we clarified that whether MPP^+^-mediated ferroptosis was modulated by ATF4, incremental GSH and reduced L-ROS were found in si-ATF4 cells treated with MPP^+^ (Fig. 2I-J, Figure S2B, Figure S2C), while overexpression of ATF4 showcased the opposite effects (Fig. 2K, Figure S2D, Figure S2E).

### ATF4 facilitated M1 polarization in BV2 cells treated by MPP^+^

**Fig. 3:**
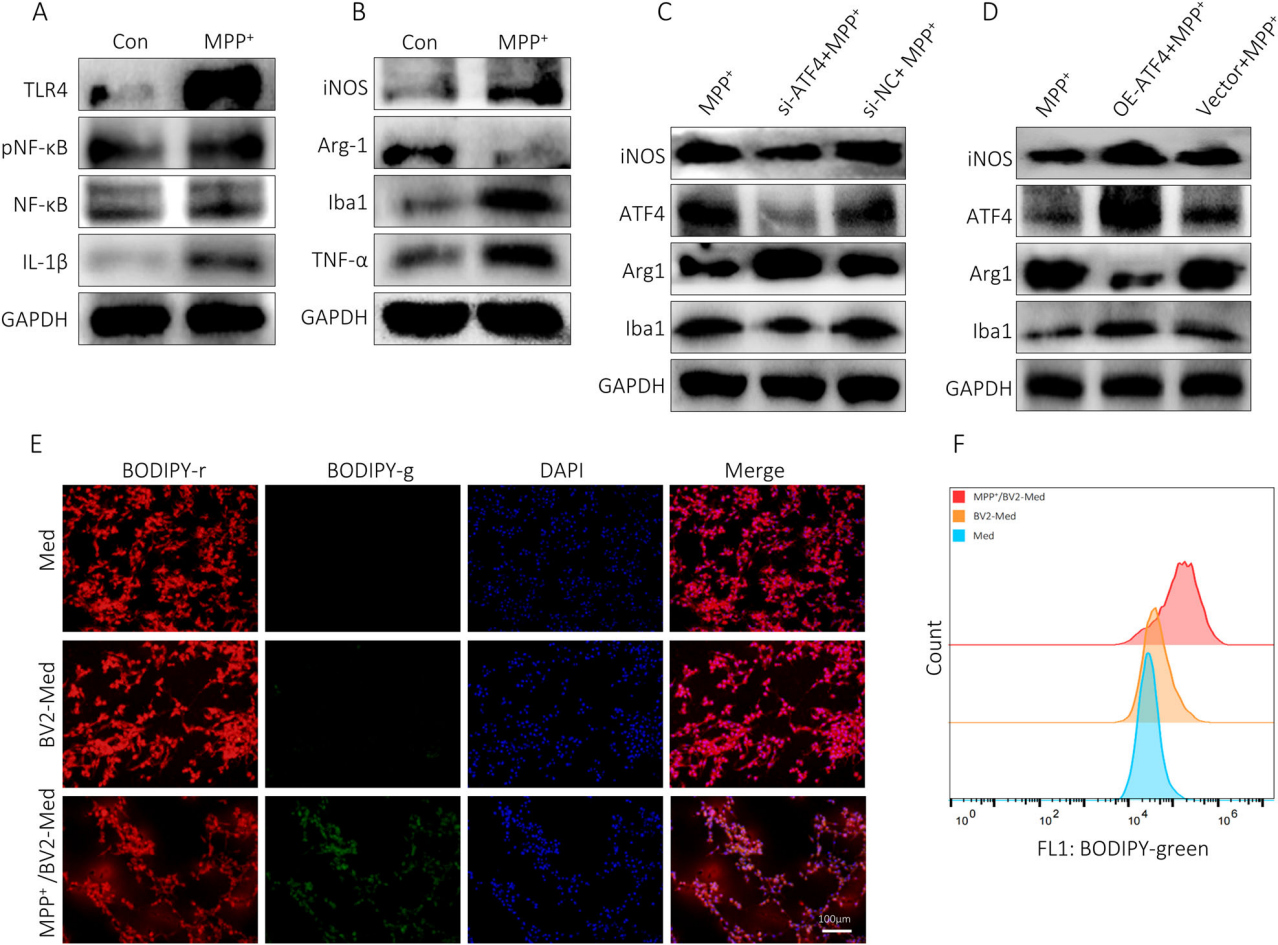
ATF4 facilitated M1 polarization in BV2 cells treated by MPP^+^. **A**, **B** Expressions of M1 phenotype markers by western blot. **C** Inhibition of ATF4 attenuated M1 phenotype. **D** Exogenous ATF4 augmented M1 phenotype. **E**, **F** Immunofluorescence and flow cytometry results of PC12 cells incubated for 24 h in media from BV2 cells treated with or without MPP^+^.

Microglia can be stimulated by LPS to pro-inflammatory (M1) phenotype for expression of pro-inflammatory cytokines [13]. Here, activated BV2 cells exhibited a shift towards M1 phenotype as confirmed by increased iNOS, TNF-α, IL-1β, Iba1, pNF-κB, and TLR4 and decreased Arg-1 after MPP^+^ treatment (Fig. 3A, B). To deeper probe the role of ATF4 on polarization of BV2 cells, we utilized siRNA to successfully reduce ATF4 expression, our findings revealed inhibition of ATF4 relented M1 phenotype of MPP^+^-treated BV2 cells with significant downregulation of iNOS and Iba-1, upregulation of Arg-1 (Fig. 3C). On the contrary, MPP^+^ induced-M1 was apparently strengthened in BV2 cells with exogenous expression of ATF4 (Fig. 3D). The supernatant of 100 μm/ml MPP^+^-treated BV2 cells was used as conditioned medium (CM), increased levels of IL-1β and TNF-a have been confirmed by ELISA (Figure S3A, B), PC12 cells were co-culture with CM for 24 h. Treatment with CM significantly increased L-ROS levels in PC12 cells (Fig. 3E, F, Figure S3C).

### ATF4 enhanced neuroinflammatory of BV2 cells via TLR4/NF-κB

**Fig. 4:**
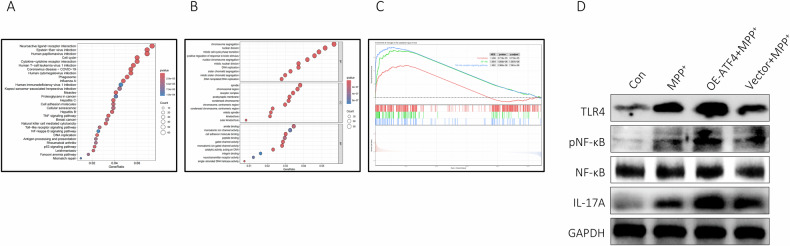
ATF4 augmented neuroinflammatory of BV2 cells via TLR4/NF-κB. **A**, **B** KEGG and GO analysis of DEGs in microglia of MPTP-induced mice. **C** Results of GSEA on DEGs in microglia of MPTP-induced mice. **D** Expression of TLR4, NF-κB, pNF-κB and IL-17A by western blot.

Compelling evidence demonstrated that ferroptosis played a vital role in inflammation. In this study, RNA-seq data of microglia from SN of mice subjected to MPTP were extracted from GEO database (GSE213100), results of GO and KEGG analysis revealed that DEGs mainly enriched in inflammation, immune and cell cycle (Fig. 4 A, B). Subsequently, we performed GSEA on the DEGs using 564 FRGs, 105 genes associated with NF-kB signaling pathway and 100 genes related to TLR signaling pathway (https://www.genome.jp/kegg). These results advocated that upregulated DEGs were remarkably enriched in these three pathways (Fig. 4C). Taking into account the critical role of TLR4/NF-κB signaling pathway in SN of PD, it remained unclear whether ATF4 overexpression activated BV2 cells via this pathway. The Western blot results indicated that in BV2 cells overexpressing ATF4, the expression levels of TLR4, pNF-κB, and IL-17A were all upregulated (Fig. 4D).

### E2 abated ATF4 and protected MPP^+^-treated BV2 cells via TLR4/NF-κB

**Fig. 5:**
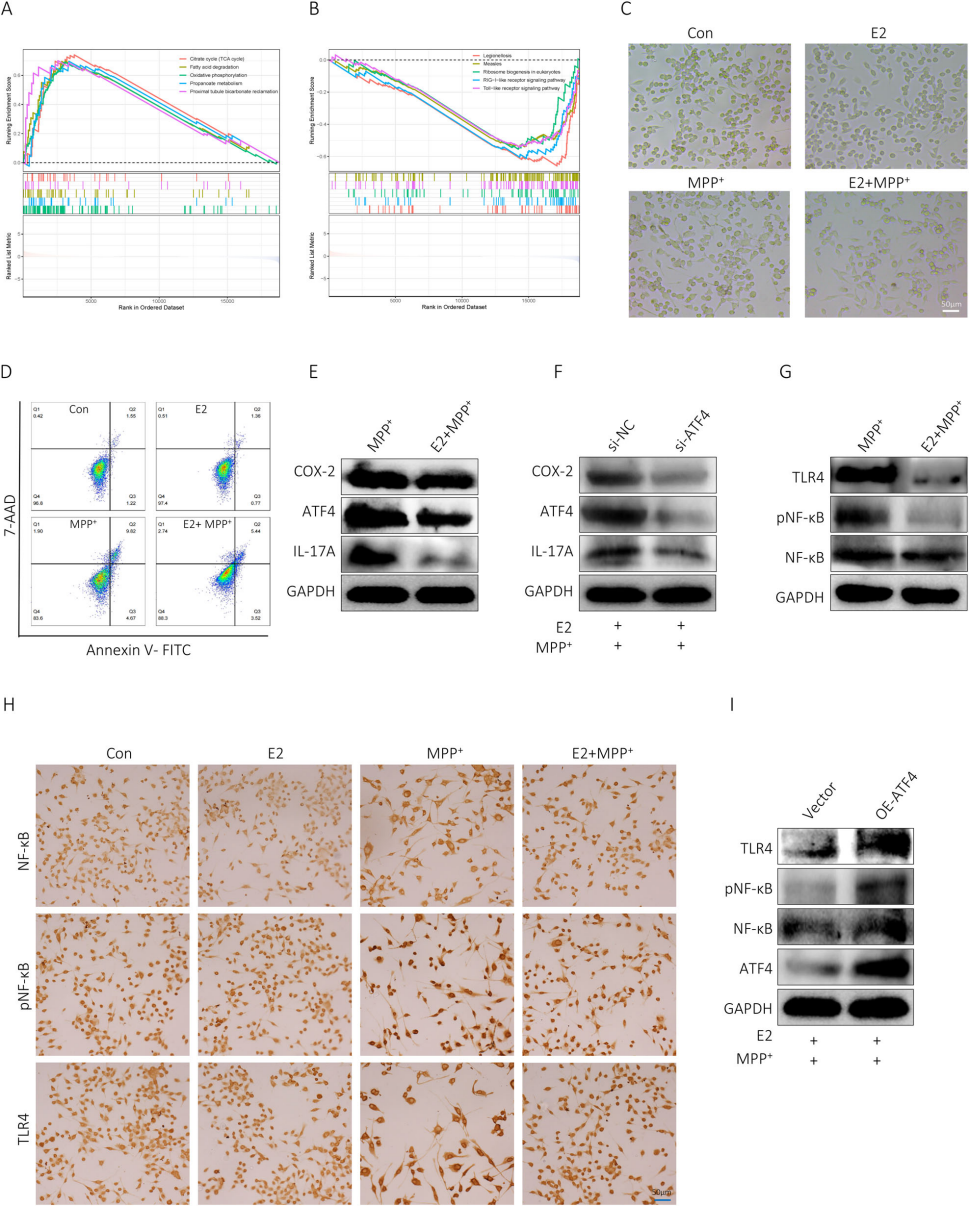
E2 downregulated ATF4 and protected MPP^+^-treated BV2 cells. **A**, **B** GSEA results based on the KEGG database for microglia from hippocampi of female and male mice treated with LPS. **C** Morphological changes of BV2 cells. **D** The ferroptosis was measured by flow cytometry using annexin V-FITC/7ADD staining. **E** Western blot showed the effects of E2 on COX-2, ATF4, and IL-17A levels in MPP^+^-treated BV2 cells. **F** Western blot showed the effects of ATF4 silencing on COX-2, ATF4, and IL-17A levels in MPP^+^ and E2-treated BV2 cells. **G** Western blot showed the effects of E2 on TLR4 and pNF-κB levels in MPP^+^-treated BV2 cells. **H** IHC confirmed TLR4 and pNF-κB expression in BV2 cells. **I** Western blot showed the effects of ATF4 overexpression on TLR4 and pNF-κB levels in MPP^+^ and E2-treated BV2 cells.

Expression programs of microglia in hippocampus of mice treated with TLR4 agonist LPS were analyzed in GEO database (GSE99622), results suggested that DEGs in microglia of male mice were significantly enriched towards inflammation and immunity (Fig. 5A, B). Given neuroprotective effects of estrogen, the role of E2 in BV2 cells were examined. E2 significantly rescued morphological changes of BV2 cells with recovered spherical or eye-shape and increased cell number (Fig. 5C), E2 significantly impeded damage of MPP^+^ on BV2 cells (Fig. 5D). Previous studies confirmed that E2 displayed adequate protection by suppressing neuroinflammation [14]. Expression profiles of microglia from hippocampal tissues of mice treated with LPS utilizing dataset GSE99622 were analyzed, pathways related to inflammation, immune response and oxidative stress exhibited downregulation in female mice compared to male mice (Fig. 5A, B). Here, after E2 intervention, ATF4 was reduced in MPP^+^-processed BV2 cells, COX-2 and IL-17A were significantly decreased (Fig. 5E). ATF4 knockdown scaled up protective function of E2 on MPP^+^-treated BV2 cells, as confirmed by decreased COX-2 and IL-17A (Fig. 5F). E2 downregulated TLR4, inhibited NF-κB phosphorylation and nuclear translocation (Fig. 5G, H), ATF4 overexpression deteriorated inhibition of E2 on pNF-κB and TLR4 in MPP^+^-treated BV2 cells (Fig. 5I).

### E2 inhibited ferroptotic stress and M1 transition of BV2 cells via ATF4

**Fig. 6:**
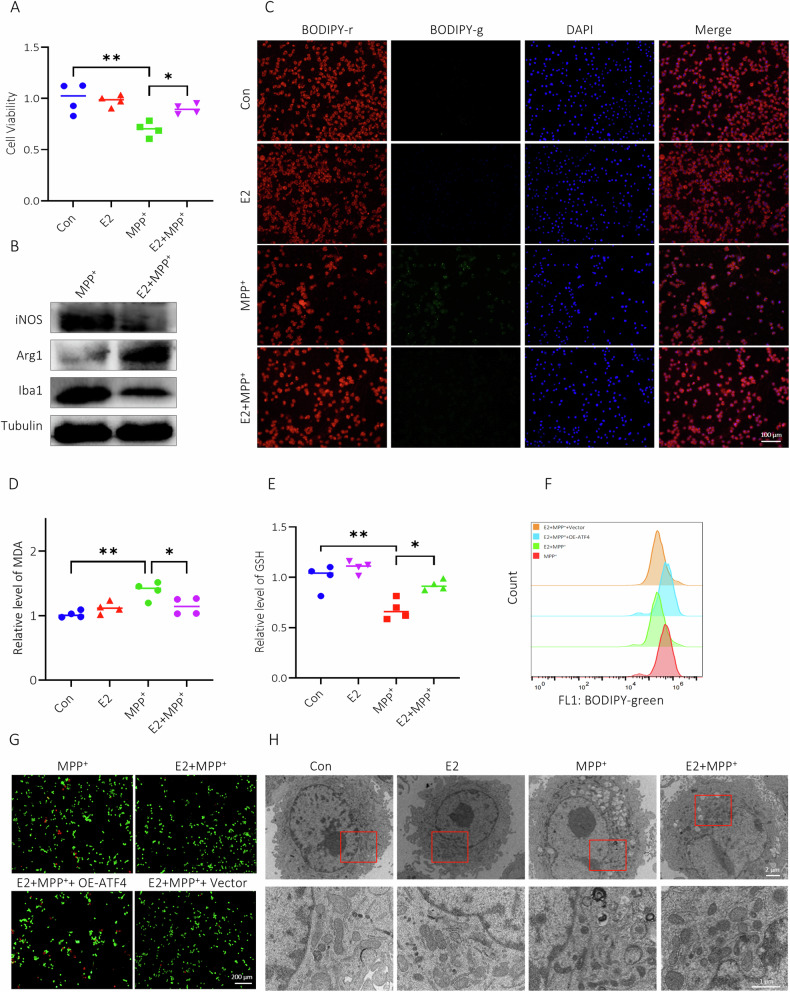
E2 inhibited ferroptotic stress and M1 transition of BV2 cells via ATF4. **A** Cell viability results of BV2 cells treated with different conditions. **B** Influence of E2 on expressions of M2- and M1-related markers in BV2 cells. **C** E2 reduced L-ROS levels in BV2 cells treated with MPP^+^. **D**, **E** Level of MDA and GSH in BV2 cells. **F** L-ROS levels in BV2 cells. **G** The live/dead assay of BV2 cells (the green fluorescence stands for living cells and the red fluorescence represents dead cells). **H** TEM images of the ultrastructure in BV2 cells from each treatment group. *P < 0.05, **P < 0.01.

Results CCK-8 assay exhibited that E2 significantly augment cell viability of MPP^+^-processed BV2 cells (Fig. 6A), moreover, E2 weakened M1 phenotypic polarization trend in BV2 cells markedly (Fig. 6B). Results of L-ROS staining showed that E2 significantly reduced L-ROS levels in MPP^+^-treated BV2 cells (Fig. 6C). As shown in Fig. 6D and E, E2 notably improved the decreased GSH levels and increased MDA levels in BV2 cells that had been caused by MPP^+^ treatment. Administration of E2 significantly downregulated ATF4 expression in MPP^+^ -treated BV2 cells (Fig. 5E). Overexpression of ATF4 reversed the protective role of E2 on MPP^+^ -treated BV2 cells by elevating L-ROS levels (Fig. 6F, Figure S4A) and increasing cell death (Fig. 6G). TEM results showed shrunken mitochondria with increased membrane densities in MPP^+^-treated BV2 cells; these effects were alleviated with E2 treatment (Fig. 6H).

### E2 ameliorated biological behavior of PD mouse

**Fig. 7:**
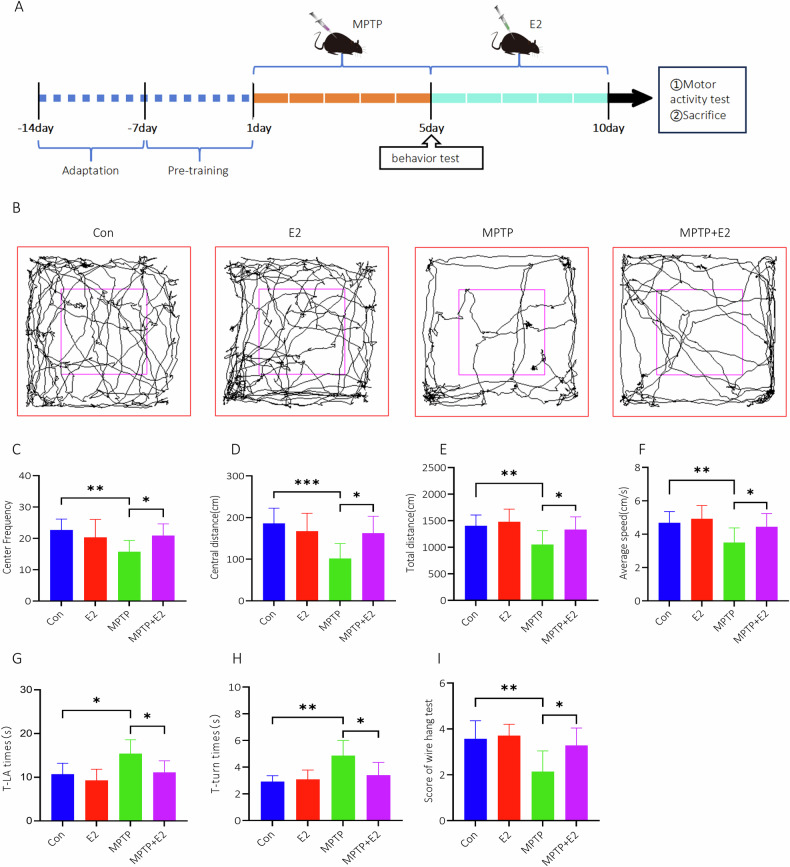
E2 ameliorated biological behavior. **A** Schematic representation of the experimental timeline. **B** Representative movement trajectories of mice in the open field test under different treatment conditions: Control (Con), E2, MPTP, and MPTP + E2. **C** Center frequency, (**D**) Central distance traveled (cm), (**E**) Total distance traveled (cm), and (**F**) Average speed (cm/s) of mice in each group. **G**, **H** Behavioral changes were assessed using pole test. **I** Behavioral changes were tested using wire hang test. *P < 0.05, **P < 0.01.

Mice were subjected to an open field test 1 day before modeling, and then 5 and 10 days after the injection of MPTP (Fig. 7A). Significant reductions in the movement path, total distance, traveled distance in center square and times of entering center were observed in PD group, the distance traveled and number of entries in the zone center were increased in MPTP + E2 group (Fig. 7B–F). Behavioral assessments from pole tests indicated that MPTP-injected mice took significantly longer to accomplish the trial compared to non-MPTP-injected mice, PD mice subjected to E2 performed better (Fig. 7G, H). Wire hang tests were employed to measure muscle strength, MPTP-treated PD mice exhibited a significant reduction in hanging scores, PD mice subjected to treatment of E2 showed a notable improvement in wire hang test scores (Fig. 7I).

### E2 relieved inflammatory via modulating ATF4 and NF-κB in vivo

**Fig. 8:**
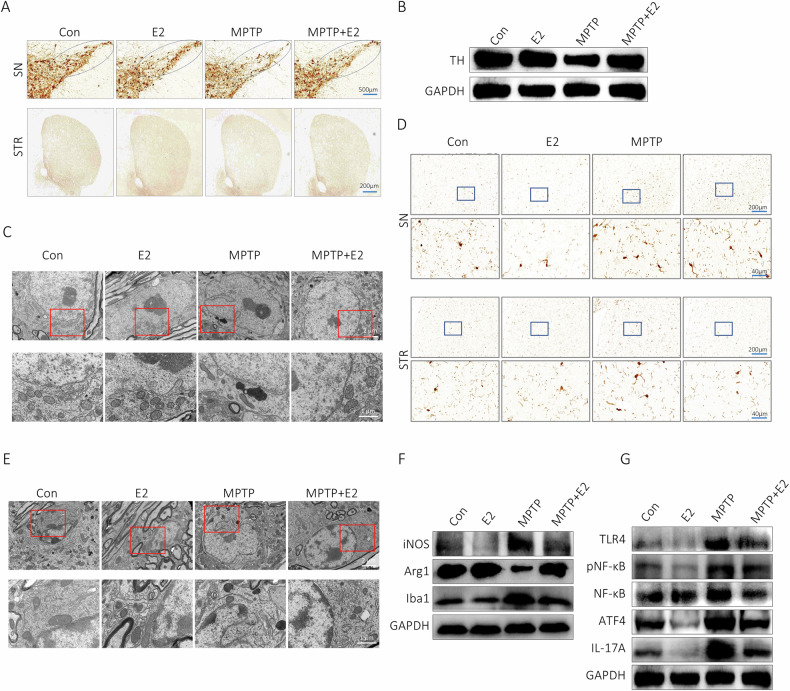
Protective effects of E2 on DANs death in MPTP mouse model. **A** Immunohistochemical results of TH in SN and STR. **B** TH expression in STR by western blot. **C** TEM images of neurons in SN. **D** Immunohistochemical results of Iba-1 in SN and STR. **E** TEM images of microglia in SN. **F** Expressions of Iba1, iNOS and Arg1 in STR by western blot. **G** Expressions of ATF4, IL-17A and pNF-κB in STR by western blot.

Expression of TH was decreased significantly in SN and STR of PD animal model, and the number of TH positive cells decreased in SN; TH-positive neurons in SN were markedly increased after E2 treatment (Fig. 8A, B). After exposure to MPTP, the neurons in SN displayed nucleus shrank out of shape, swollen and incomplete mitochondria and Golgi apparatus, these damages were improved after treatment with E2 (Fig. 8C). Previous studies have established that microglia play a critical role in inflammation during the onset and progression of PD. We further examined microglia in the SN and STR of mice. Our results demonstrated that MPTP treatment significantly activated microglia in both the SN and STR of mice, characterized by an increase in microglial volume and number. This activation was notably ameliorated by E2 treatment (Fig. 8D). Electron microscopy results corroborated that E2 rescued MPTP-induced microglial damage in the SN, shrunken mitochondria was observed in MPTP group, moreover, E2 treatment alleviated mitochondria injuries (Fig. 8E). Additionally, western blot analysis confirmed that E2 reduced expressions Iba1 and iNOS and augmented expression of Arg1 in STR, these results suggested that E2 inhibited MPTP-induced polarization of microglia to M1 phenotype and promoted polarization to M2 phenotype (Fig. 8F). To investigate whether E2-mediated inhibition of microglial activation involved in vivo regulation of ATF4, we assessed ATF4 expression in STR of PD mouse models. In the model, E2 downregulated ATF4, IL-17A and pNF-κB levels (Fig. 8G).

## Discussion

Over the past 20 years, the global burden of PD patients has nearly doubled, and this increase will continue as the aging process intensifies [15]. Several salient and well established features of PD are consistent with ferroptosis [2, 16, 17]. By performing RNA sequencing analysis on GEO dataset, we found differentially expressed gene profile was enriched in ferroptosis displayed by GSEA, moreover, ATF4 was one of the most DEGs in PD patients. ATF4 was also increased in microglia and dopaminergic neurons (DANs) in rat PD model induced by rotenone [18].

ATF4 is implicated in regulation of iron metabolism and response to oxidative stress which are critical components of ferroptosis. Previous research has established contradictory function of ATF4 in pathogenesis of neurodegenerative diseases. ATF4 can be upregulated in PD as a response to neuronal stress, contributing to pathogenesis by affecting protein folding and aggregation [19]. A previous study suggested that ATF4 has a cytoprotective effect during the treatment of PC12 cells [20]. On the contrary, a cytotoxic effect of continuously overexpressed ATF4 exhibited PD-like pathology in animals [19]. So, ATF4 may either promote or inhibit ferroptosis depending on cellular context and balance of stress signals [21].

In view of ATF4 ectopically expressed in STR/SN of PD patients, here, ATF4 was determined in MPP^+^-induced BV2 cells, we found that ATF4 was increased in MPP^+^-induced BV2 cells or SN of MTPT-treated mice, upregulated ATF4 was coincided with our bioinformatics analysis. We further reported a relationship between ATF4 and inflammatory mediators in microglial cell treated by MPP^+^. Knockdown of ATF4 weakened ferroptotic stress of BV2 cells induced by MPP^+^, as confirmed by significant downregulation of L-ROS and decreased cell death number. On the contrary, ATF4 overexpression uplifted MPP^+^-induced activation of BV2 cells. Together, these data indicated that ATF4 regulated ferroptosis and lipid peroxidation in pathogenesis of PD.

Abnormal and continuous activation of astrocytes and microglia promoted expressions of proinflammatory cytokines and lead to deformation and dysfunction of DANs [22]. In this study, a GEO cohort of MPTP-induced PD mouse model was used for KEGG analysis, enriched pathways were involved in inflammation, such as TNF. NOD−like receptor, and TLR signaling pathways. In our experiment, MPP^+^ significantly increased iNOS, IL-1β and TNF-α in microglia in vitro; expressions of IL-17A and iNOS in midbrain of PD mice were also increased. Ferroptosis inhibitor or inhibition of ATF4 suppressed overproduction of proinflammatory cytokines in MPP^+^-treated BV2 cells.

Growing evidences indicated TLR4/NF-κB-mediated neuroinflammation played an important role in pathogenesis of PD [23, 24]. Our bioinformatics analyses revealed that pronounced activations of inflammation and immune pathways were present in PD patients and PD animal models. TLR4 led to decreased activity of DANs and inhibited expression of TH in ventral tegmental area [25]. Clinical trials found that abnormally increased TLR4 in serum of PD patients was closely related to progression of PD [23]. In previous research, it is found that activated ATF4 induce macrophages polarized to M1 phenotype which overexpress TLR4 [26, 27]; here, by constructing PD mouse model, we found increased TLR4 in STR of PD mice, TLR4/NF-κB pathway was surprisingly attenuated in ATF4 silenced BV2 cells treated with MPP^+^, accompanied by decreased IL-17A and COX-2, indicating that ATF4 promoted microglia-mediated neuroinflammation through activating TLR4/NF-kB pathway.

We extracted expression profiles of microglia from hippocampi of both male and female mice treated with LPS from GSE99622, microglia of male mice demonstrated a markedly higher upregulation of gene expressions related to inflammatory and immune pathways. An increasing body of evidence found that E2 regulated inflammation, oxidative stress [28], neuron-derived E2 was neuroprotective and momentous in hastening reaction of astrocytes which produced astrocyte-derived neurotrophic factors [29]. In this study, we found E2 restrained ATF4 in MPP^+^-treated cells, inhibition of ATF4 augmented protective effects of E2 with decreased L-ROS and proinflammatory cytokines. Furthermore, E2 inhibited activation of microglia induced by MPTP and reduced production of iNOS and IL-17A, E2 significantly inhibited phosphorylation of NF-κB and reduced expression of TLR4 in vivo. Regarding behavioral signs of PD mouse model, the T-total time and T-turn time in MPTP-induced PD mice were elongated. Compared to MPTP group, the T-turn time and total time of PD mice treated with E2 were significantly reduced in the polarity test, and the traction test score was significantly increased. E2-treated PD mice displayed a demonstrably amelioration in wire hang performance.

In conclusion, we verified that E2 protected BV2 cells from MPP^+^-induced injury, inhibited activation of microglia, and increase number of TH positive cells in SN of PD mice. Further investigation suggested that E2 could well inhibit generation of pro-inflammatory cytokines driven by TLR4/NF-κB pathway via regulating ATF4 which might lead to better and complimentary therapy to PD.

## Materials And Methods

### Bioinformatics analysis

Gene expression profiles (GSE7621, GSE20292, GSE68719, GSE109329, GSE152100 and GSE99622) were downloaded from GEO database (http://www.ncbi.nlm.nih.gov/geo). The GSE7621 cohort included 25 post-mortem substantia nigra (SN) samples, with 16 from idiopathic PD patients and 9 from age-matched controls without PD. GSE109329 data included transcriptome analysis results of mouse microglia under 96 different in vitro stimulus conditions, MPP^+^ treatment group within the dataset was analyzed. The most core and potential key genes were validated by GSE20292, GSE68719, GSE152100 gene sets. Differentially expressed genes (DEGs) between control specimens and PD was screened using Limma package (version 4.3.2), where *P*-values were adjusted for multiple testing using Benjamini-Hochberg (BH) method. Only the genes with FDR < 0.05 and |log2 fold change (FC) | > 1, as a commonly used criteria, were selected as DEGs. The Kyoto Encyclopedia of Genes and Genomes (KEGG) pathway enrichment was analyzed with ‘clusterProfiler’ R package. Ferroptosis-related genes (FRGs) were sourced from FerrDb database (http://www.zhounan.org/ferrdb/). “WGCNA” R package was used to execute weighted gene co-expression network analysis (WGCNA), lasso regression analysis was performed using “glmnet” R package, random forest analysis was carried out using “randomForest” R package, and protein-protein interaction (PPI) analysis was performed using “STRINGdb” R package. Enrichment of biological functional categories were identified by gene set enrichment analysis (GSEA) using “clusterProfiler” and “enrichplot” R package based on GSE99622 cohort.

### Cell culture and transfection

BV2 and PC12 cells sourced from Shanghai Cell Bank of the Chinese Academy of Sciences (Shanghai, China) was maintained in RPIM-1640 medium (Hyclone, Logan, UT, USA) containing 10% FBS (Hyclone; Logan, UT, USA), 100 U/mL of penicillin, and 100 μg/mL streptomycin (Solarbio, Beijing, China) at 37 °C in a humidified atmosphere containing 5% CO_2_. Cells were pretreated with 10 nM E2 (ThermoFisher, Waltham, MA, USA) or without it for 1 h before being stimulated with 100 μM MPP^+^ (Sigma-Aldrich, St. Louis, MO, USA) for 24 h. Bv2 cells were infected with lentivirus-mediated knockdown of Atf4 (lentiAtf4-shRNA) or a control non-coding (NC) lentiviral system purchased from Genepharma (Shanghai, China). pCDNA3.1-mATF4 (Catalog #:21845; Addgene) or pCDNA3.1 were transfected using LipofectamineTM3000 (ThermoFisher, Waltham, MA, USA) according to manufacturer’s protocol.

### Flow cytometry (FCM)

In total 3 × 10^5^ BV2 cells were inoculated in 6-well plates for 24 h, then collected cells were dyed with Annexin V-FITC/7AAD kit (Beckman Coulter, Marseille, France) after treatment. The specimens were detected by FCM (Becton Dickinson, San Jose, CA, USA). Finally, cell ferroptosis ratio was analyzed via ACEA NovoCyte (ACEA Pharma; San Diego, CA, USA).

### Measurement of lipid reactive oxygen species(L-ROS)

In total 1 × 10^5^/well BV2 cells were planted in 6-well plates. The drug treated-cells were dyed by 10 μM C11-BODIPY 581/591 (ThermoFisher, Waltham, MA, USA) for 30 min. The fluorescence emission peak shifted from 590 nm to 510 nm after oxidation of polyunsaturated butadiene portion of C11 BODIPY was detected utilizing FCM or fluorescence microscope (Olympus BX53, Japan) to manifest L-ROS.

### Assessment of glutathione (GSH) and malondialdehyde (MDA)

Reduced GSH and MDA levels were determined using the GSH assay kit (Beyotime, Nanjing, China) and the MDA assay kit (Jiancheng Bioengineering, Nanjing, China), respectively, according to the manufacturer’s instructions.

### Cell counting kit-8 (CCK-8) assay, living and dead cell assay

Cell viability was determined using CCK-8 assay (Beyotime Biotechnology, Shanghai, China). A total 2 × 10^3^ BV2 cells were seeded in each well of 96-well plate and incubated for 24 h, various concentrations of drugs were administered, and the cells were incubated for an additional 24 h to evaluate drug toxicity. Absorbance at 450 nm was measured using a microplate reader to assess cell viability. Additionally, live and dead cells were distinguished using a calcine-AM/PI assay kit (Beyotime Biotechnology, Shanghai, China).

### Electron microscopy

For observation of ultrastructural changes, fresh 1 mm-thick SN/STR slices or cultured cells pellet were fixed in 2.5% glutaraldehyde overnight at 4 °C, washed three times with 0.1 M PBS, fixed in 1% osmium tetroxide for 2 h at 4 °C, then dehydrated with a graded series of ethanol and embedded in epoxy resin. Randomly selected ultrathin sections were stained with uranyl acetate and lead citrate and observed under an electron microscope (HT-7700, Hitachi, Tokyo, Japan).

### Western blot

Total proteins were extracted using protein lysate (Sangon Biotech, Shanghai, China) and separated on a 10% SDS-PAGE. The proteins were subsequently transferred to a polyvinylidene fluoride (PVDF) membrane (Millipore, Billerica, MA, USA). The membrane was blocked and incubated overnight at 4 °C with the following primary antibodies: ATF4 (Cell Signaling Technology, Danvers, MA, USA), ionized calcium-binding adaptor molecule 1 (Iba1; Genetex; Hsinchu City, Taiwan, China), IL-17A, cyclooxygenase-2 (COX-2), IL-1β (Bioss Biotechnology, Beijing, China), inducible nitric oxide synthase (iNOS), arginase 1 (Arg-1), TLR4, and tumor necrosis factor α (TNF-α) (Abcam, Cambridge, MA, USA), GAPDH, β-actin, NF-κB, pNF-κB (ProteinTech, Wuhan, China), and tyrosine hydroxylase (TH; Sigma, St. Louis, MO, USA). Then the membrane was exposed to HRP-labeled secondary antibodies (Abclonal, Wuhan, China) at room temperature for 1 h, followed by detection with an enhanced chemiluminescence (ECL) solution (Beyotime Biotech, Nanjing, China) and imaged using a chemiluminescence imaging system. Band intensities were quantified using Image J software (version 1.53e; NIH, USA).

### Construction of PD mouse model and drug treatment

Specific pathogen-free (SPF) male C57BL/6 mice, aged 12 weeks and weighing 25–30 g, were obtained from Vital River Laboratory Animal Technology (Beijing, China). The experimental protocols were approved by the Animal Ethics Committee of Shandong Second Medical University (approval code: 2018-130), adhering to the guidelines set forth in the National Institutes of Health Guide for the Care and Use of Laboratory Animals. To induce a PD mouse model, mice received daily intraperitoneal injections of MPTP dissolved in saline (30 mg/kg/day) for 5 consecutive days. The animals were randomly assigned to four groups (*n* = 12 per group): control, MPTP, E2, and MPTP + E2. On day 6, the groups were administered E2 (1 μg per injection, 10 μg/mL concentration) or an equivalent volume of saline twice daily for 5 days. Behavioral assessments were conducted on day 11, followed by collection of mouse midbrain or STR for subsequent western blot analysis and immunohistochemical studies.

### Animal behavioral evaluation

For open-field test, mice were placed at the center of an open-top box measuring 45 × 45 × 40 cm, behavior was recorded for 5 min using a video camera positioned above the box at 30 frames per second. The arena was sanitized with 70% ethyl alcohol and allowed to dry thoroughly between sessions. In the week preceding the commencement of the formal experiment, all groups of mice underwent a period of acclimatization training. Data from these sessions were analyzed using Smart 3.0 video tracking system (Panlab, Harvard Apparatus, UK). Metrics such as mean velocity, total distance traveled, distance covered in the central area, and frequency of center entries were quantitatively assessed. A pole test was conducted to assess movement behavior impairments. A 2.5 cm diameter cork ball was secured at the top of a 50 cm long, 2 cm diameter vertical pole, which was double-wrapped with gauze to prevent slipping. Animals was gently placed on the spherical part with head upward. The time of mice to completely turn downwards and descend to floor was recorded. Additionally, limb functionality was evaluated using a wire hang test, where test mice were suspended by fore limbs and were required to lift hind limbs to a horizontal wire. Scoring was based on hind paw engagement: 3 points for gripping with both hind paws, 2 points with one, and 1 point for no hind paw gripping.

### Tissue preparation and immunohistochemistry

Mouse was thoroughly anesthetized before being sequentially perfused transcardially with 4% paraformaldehyde, the brain was immediately immersed in 4% paraformaldehyde for 24 h to ensure comprehensive fixation. Coronal sections of 5 μm were precisely cut using a microtome which encompassed SN, spanning from Bregma -2.70 mm to Bregma -3.88 mm, and striatum (STR), from Bregma 1.78 mm to Bregma -2.3 mm. Immunohistochemistry was performed by incubating the sections with primary antibodies against TH (1:3000), Iba-1 (1:200), NF-κB (1:500) and pNF-κB (1:500) overnight at 4 °C. This was followed by incubation with horseradish peroxidase-conjugated secondary antibodies (Zhongshan Golden Bridge Biotechnology, Beijing, China) and visual development with diaminobenzidine. The observations were conducted using the bright field channel of a fluorescence microscope (Olympus BX53, Japan).

### Statistical analysis

The results are presented as means ± SEM, statistical analyses were conducted using GraphPad Prism 8 software. One-way analysis of variance (ANOVA) and Student’s t-test were employed for comparisons between groups. ANOVA was applied for experiments involving more than two groups, followed by post-hoc tests such as the Bonferroni or Games-Howell test for multiple comparisons. A significance level of *P < 0.05 was considered statistically significant.

## Supplementary information

Supplementary figure legends

Figure S1

Figure S2

Figure S3

Figure S4

Original Western Blots

## Data Availability

All bioinformatics data analyzed in this article, including GSE7621, GSE20292, GSE68719, GSE109329, GSE152100, and GSE99622, are sourced from publicly available datasets in the GEO database.
