# Supplementary material for: 17β-estradiol alleviated ferroptotic neuroinflammation by suppressing ATF4 in mouse model of Parkinson’s disease
Source: Cell Death Discov. 2024 Dec 19;10:507. doi: 10.1038/s41420-024-02273-z (PMC11659321; doi:10.1038/s41420-024-02273-z)
Supplement: Supplementary file 1 — Supplementary figure legends [file 41420_2024_2273_MOESM1_ESM.docx]

Supplementary figure legends

Supplementary figure 1 ATF4 was identified as a potential key gene in PD by bioinformatics analysis

(S1A) Boxplot distribution of gene expression levels among various sample groups. (S1B) Boxplot displayed gene expression data after the significantly different G502 group was removed and correction was applied using the limma package. (S1C) Volcano plot of differentially expressed genes from the normalized GSE109329 dataset. (S1D) Heatmap of DEGs from GSE109329 dataset. (S1E) Soft threshold analysis suggested gene associations were maximally consistent with the scale-free distribution when β=6. (S1F) The cluster dendrogram of co-expression genes in PD. (S1G) Relationship between gene importance and Parkinson’s disease in the brown module. (S1H) Log (Lambda) value of three genes in LASSO model and the most proper log (Lambda) value in LASSO model. (S1I) PPI network for significant protein–protein interactions among genes in the brown module.

Supplementary figure 2 ATF4 accelerated MPP^+^-induced cytotoxic on BV2 cells

(S2A, B, D) Relative levels of ROS. **P<0.01. (S2C, E) Expressions of ATF4 by western blot.

Supplementary figure 3 ATF4 facilitated M1 polarization in BV2 cells treated by MPP^+^

(S3A, B) Relative levels of IL-1β and TNF-a by ELISA. (S3C) Relative levels of ROS. **P<0.01.

Supplementary figure 4 E2 inhibited ferroptotic stress and M1 transition of BV2 cells via ATF4

(S4A) Relative levels of ROS. **P<0.01.
