# Supplementary figures and images for: 17β-estradiol alleviated ferroptotic neuroinflammation by suppressing ATF4 in mouse model of Parkinson’s disease
Source: Cell Death Discov. 2024 Dec 19;10:507. doi: 10.1038/s41420-024-02273-z (PMC11659321; doi:10.1038/s41420-024-02273-z)

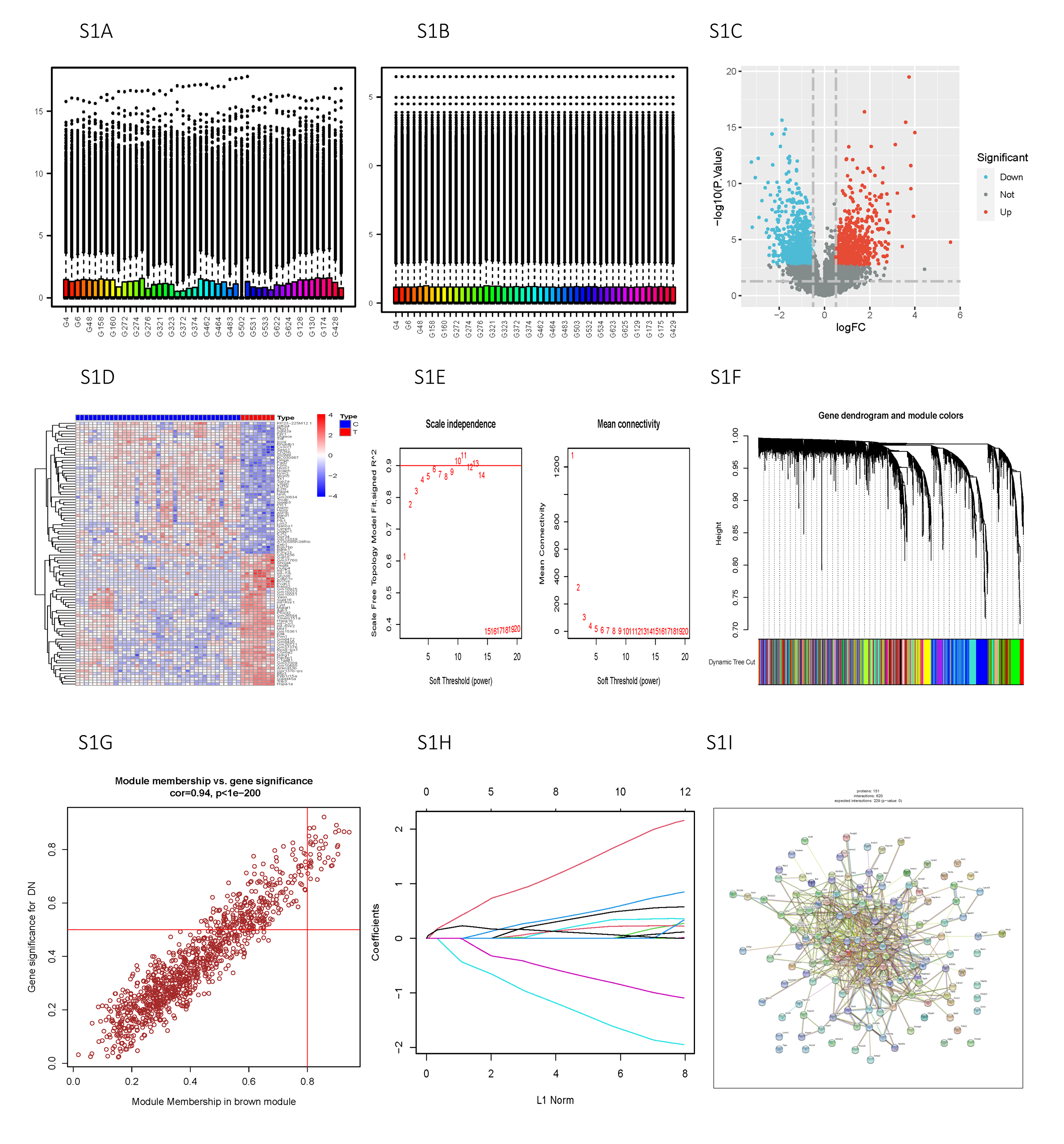

Supplement: Supplementary file 2 — Figure S1 [file 41420_2024_2273_MOESM2_ESM.tif]

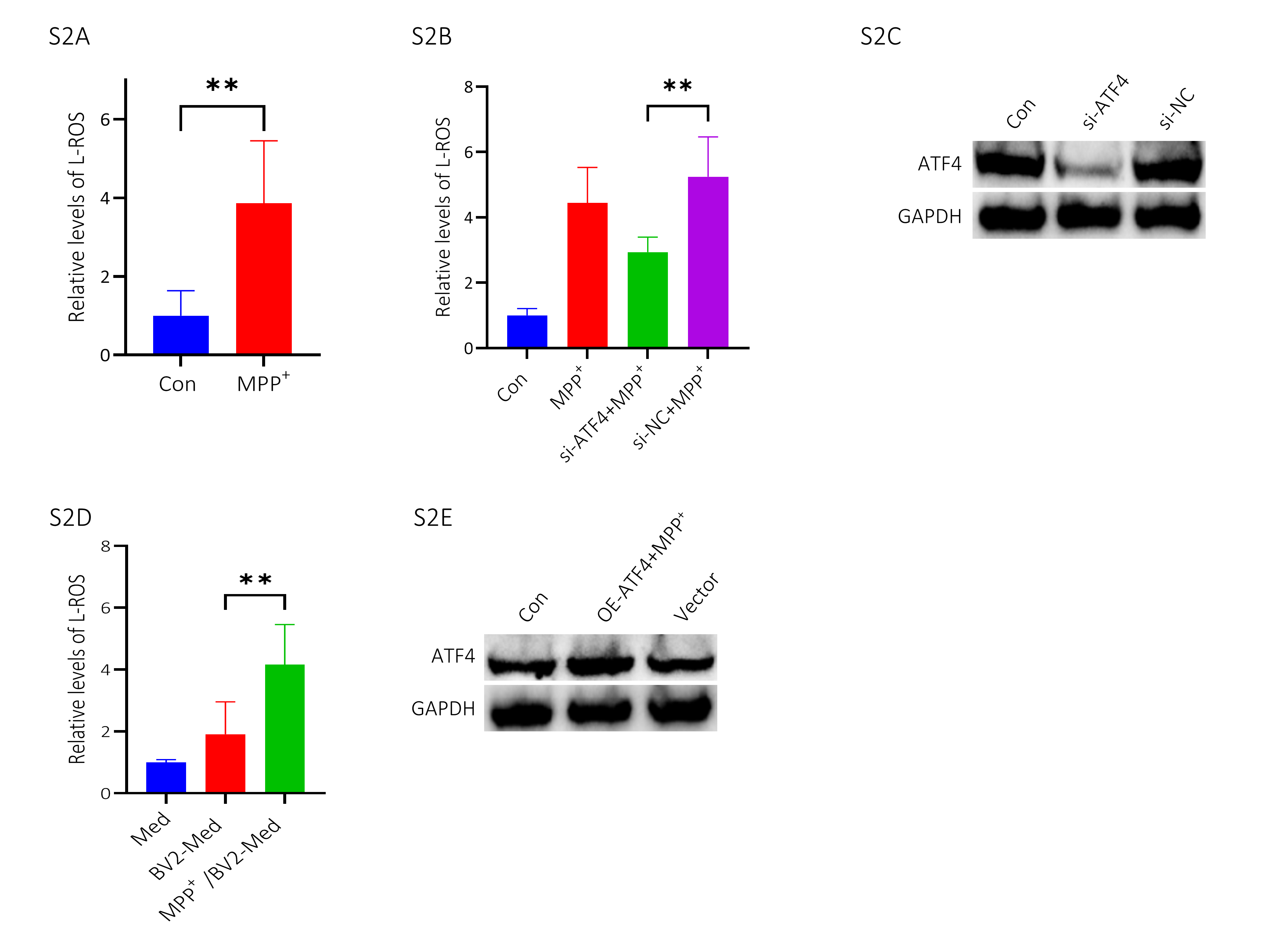

Supplement: Supplementary file 3 — Figure S2 [file 41420_2024_2273_MOESM3_ESM.tif]

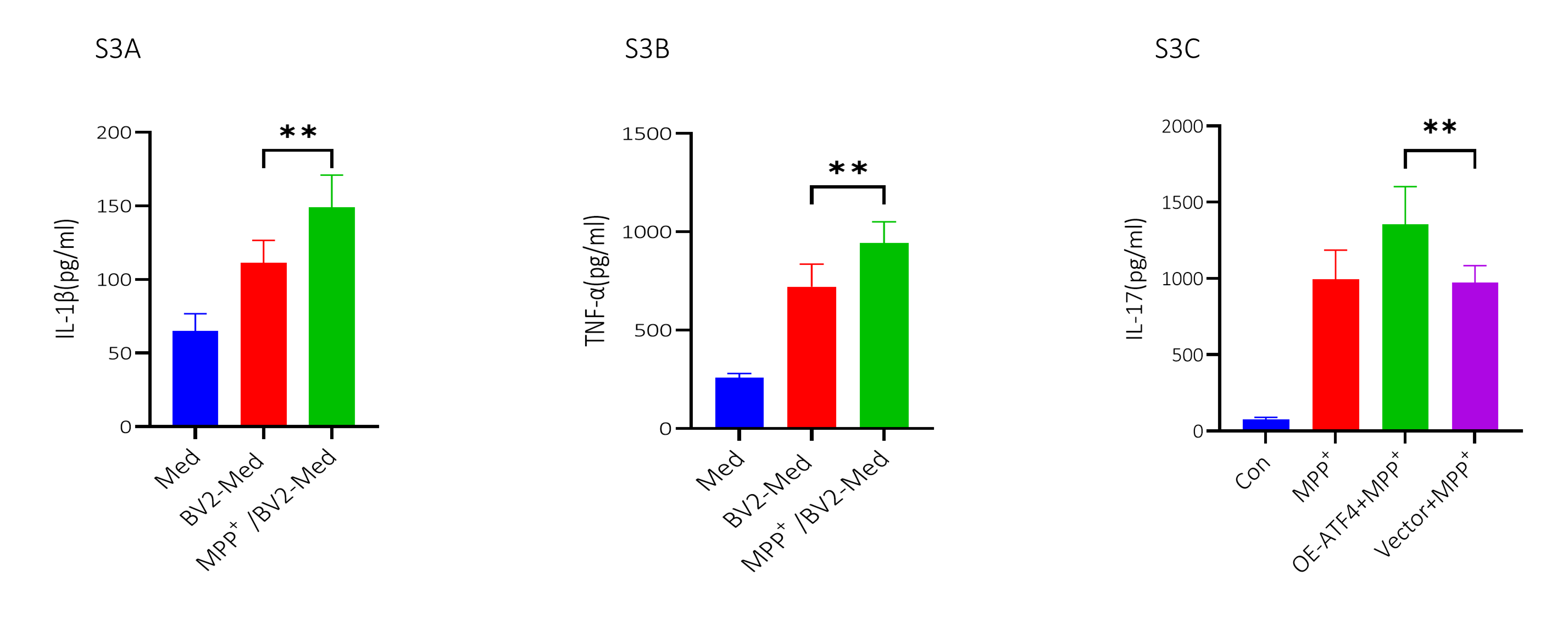

Supplement: Supplementary file 4 — Figure S3 [file 41420_2024_2273_MOESM4_ESM.tif]

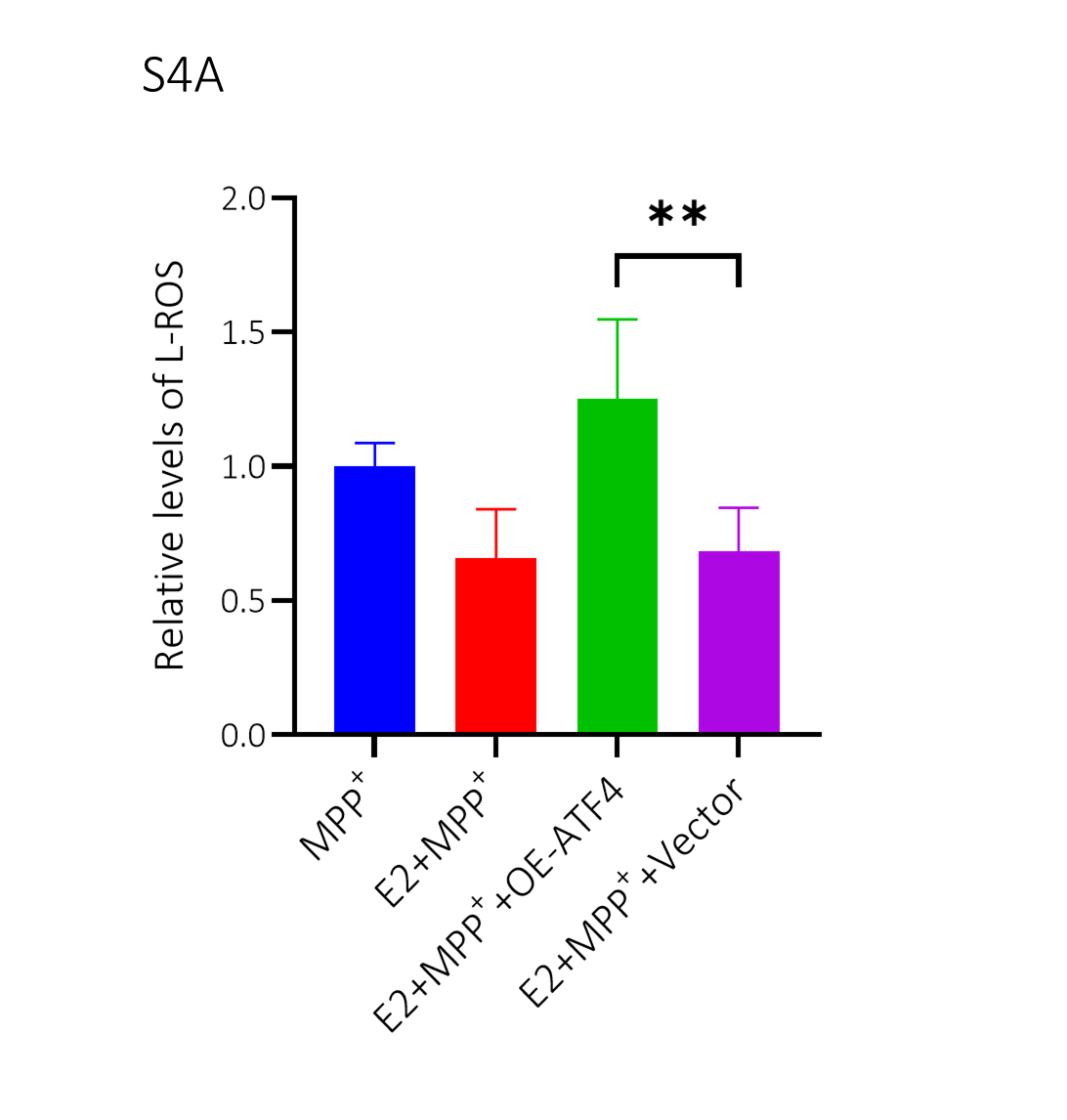

Supplement: Supplementary file 5 — Figure S4 [file 41420_2024_2273_MOESM5_ESM.tif]
