# Supplementary material for: 17β-estradiol alleviated ferroptotic neuroinflammation by suppressing ATF4 in mouse model of Parkinson’s disease
Source: Cell Death Discov. 2024 Dec 19;10:507. doi: 10.1038/s41420-024-02273-z (PMC11659321; doi:10.1038/s41420-024-02273-z)
Supplement: Supplementary file 6 — Original Western Blots [file 41420_2024_2273_MOESM6_ESM.pdf]

Supplemental Material – Original Blots

Relevant areas for cropped blots in the main and Extended Data figures are shown with **blue** boxes

Figure 2

2D

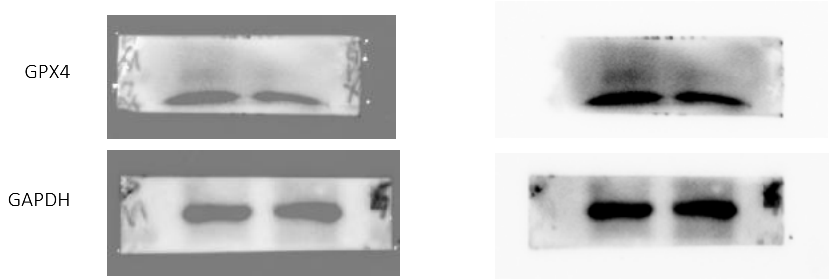

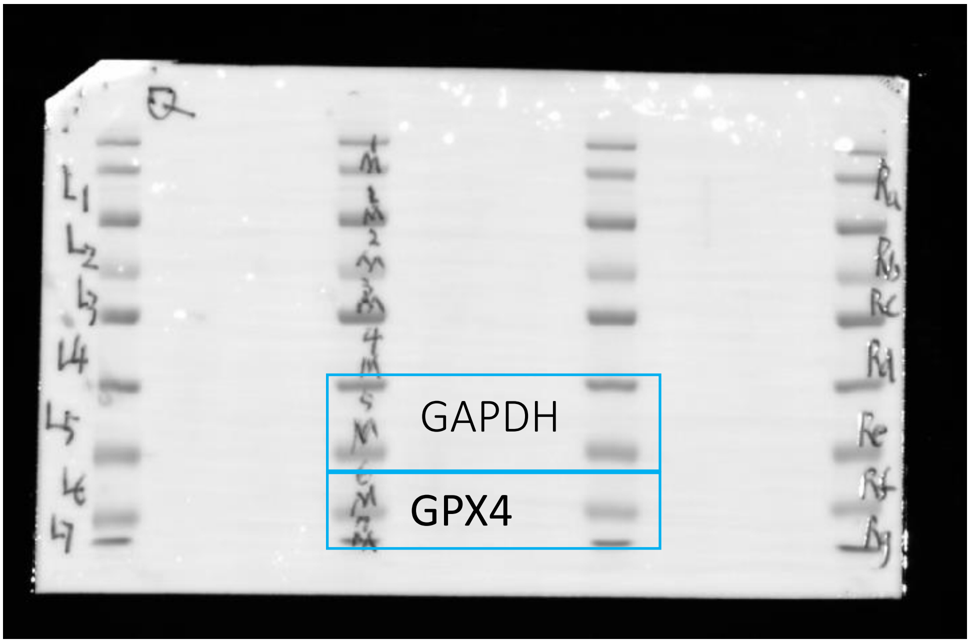

Figure S2

S2C

ATF4

GAPDH

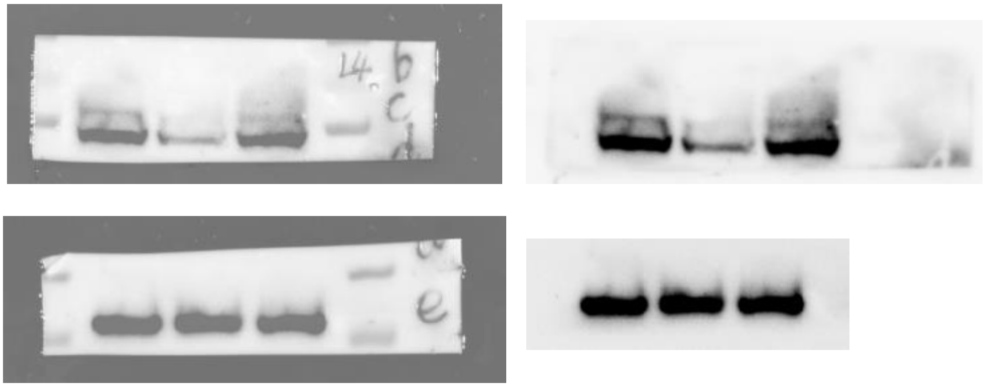

S2E

ATF4

GAPDH

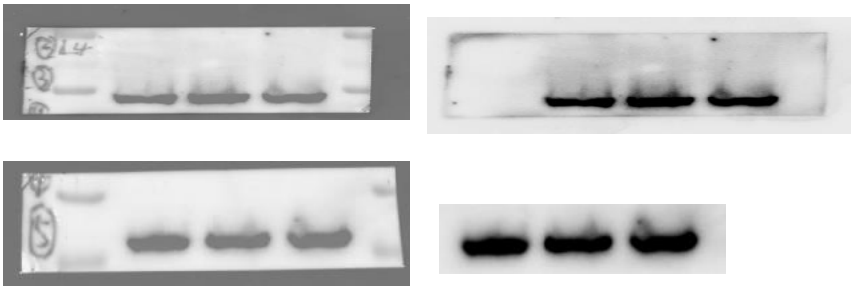

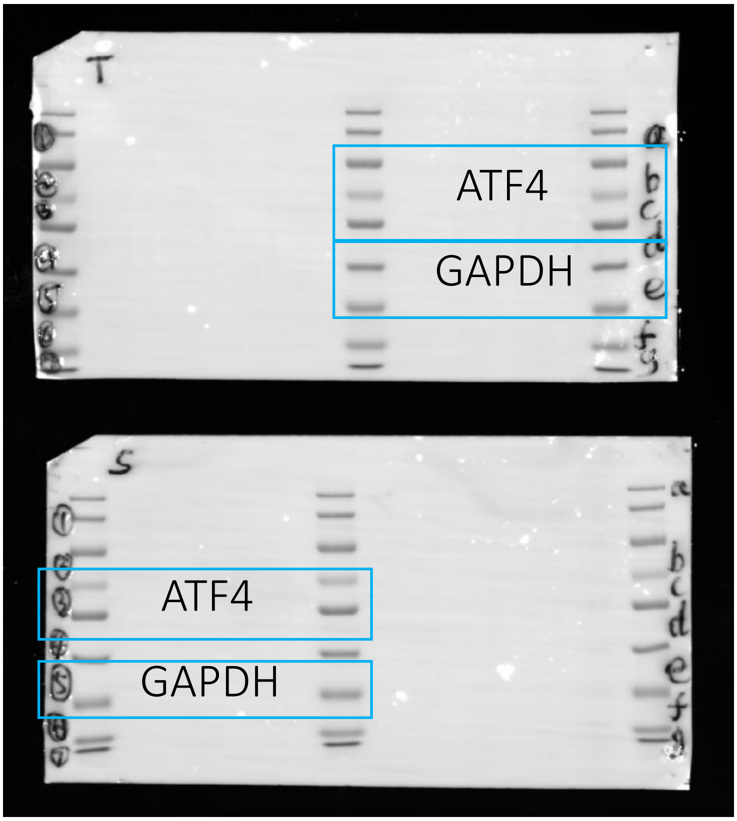

Figure 3

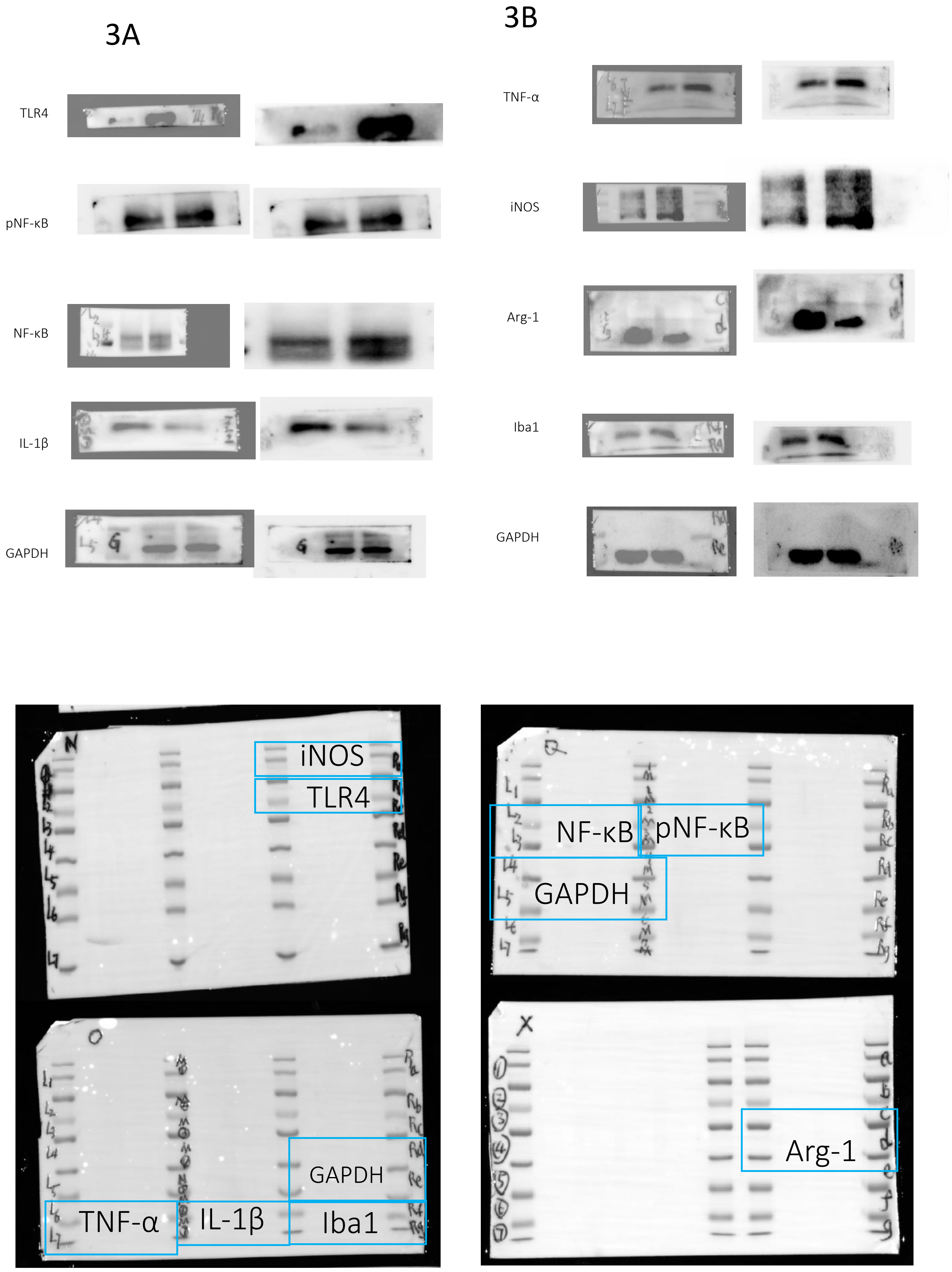

3A
TLR4
pNF-κB
NF-κB
IL-1β
GAPDH
3B
TNF-α
iNOS
Arg-1
Iba1
GAPDH
iNOS
TLR4
NF-κB
pNF-κB
GAPDH
GAPDH
TNF-α
IL-1β
Iba1
Arg-1

Figure 3

3C

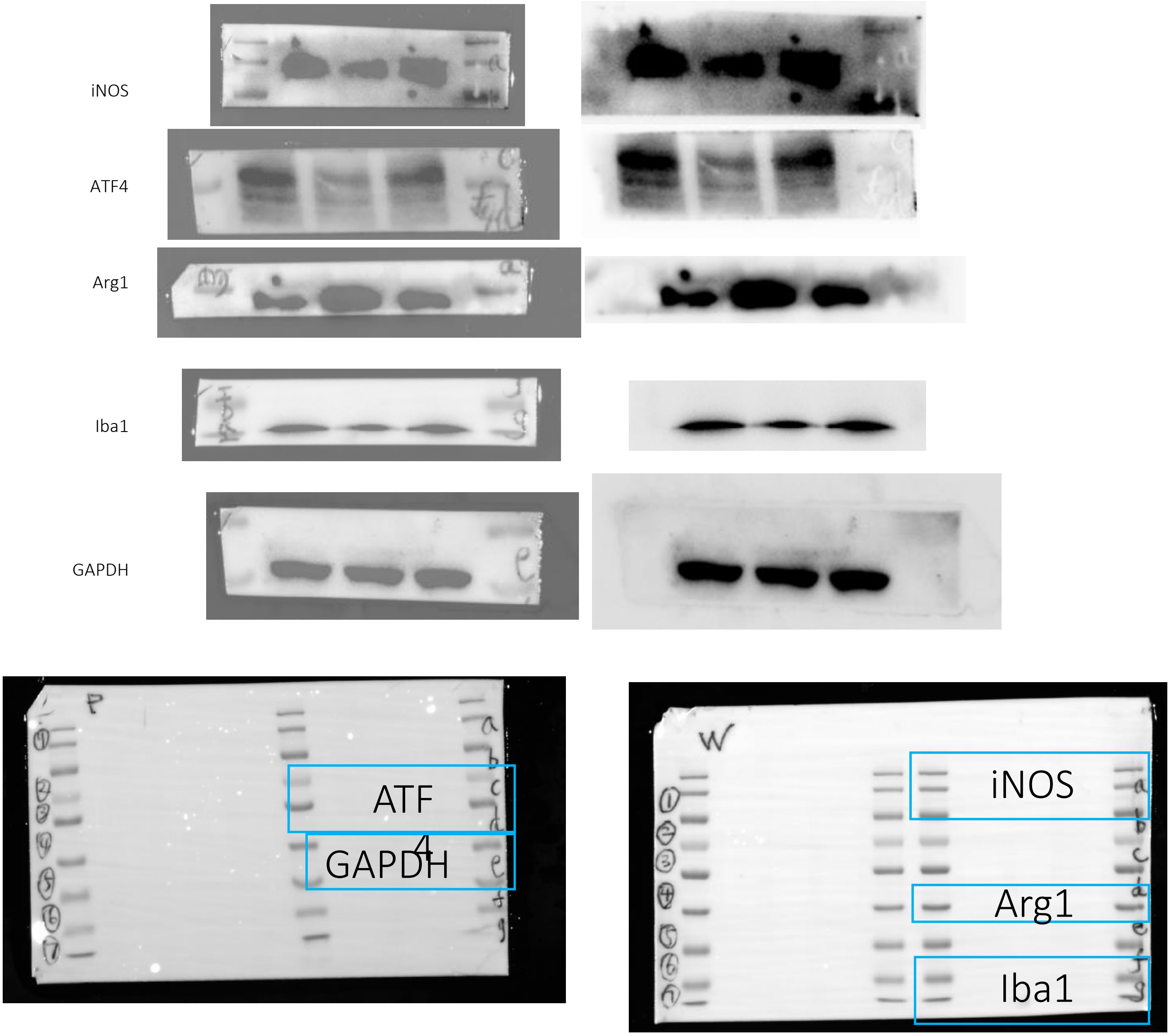

Figure 3

3D

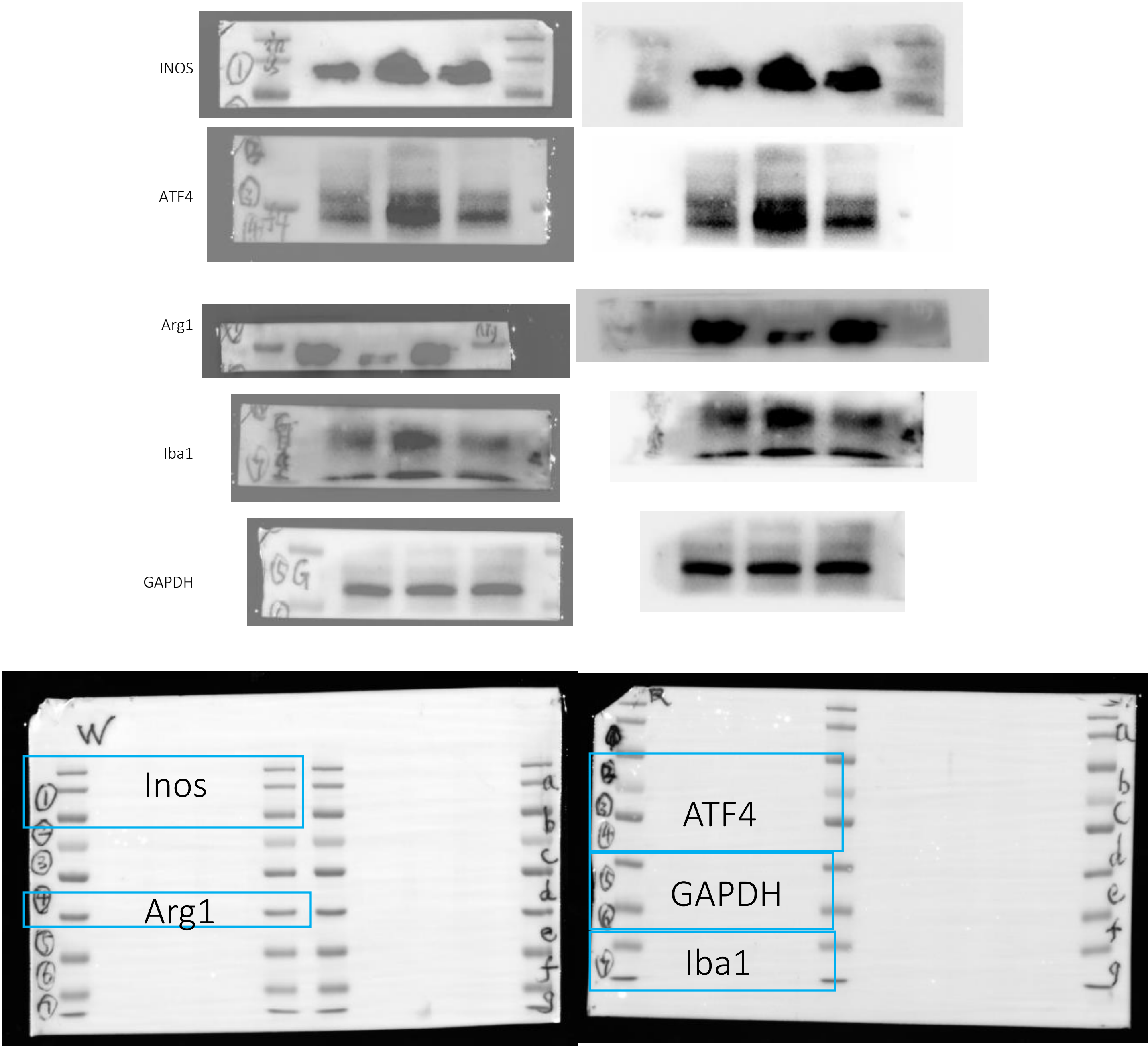

Figure 4

4D

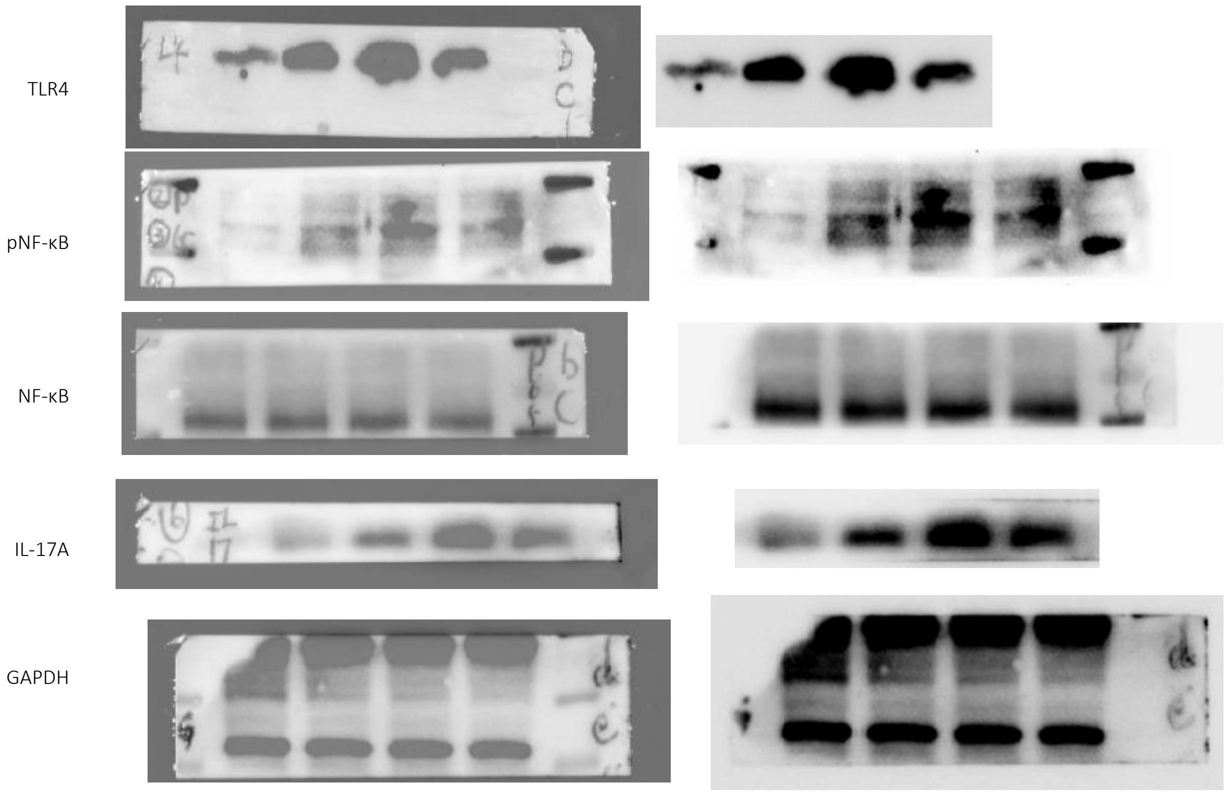

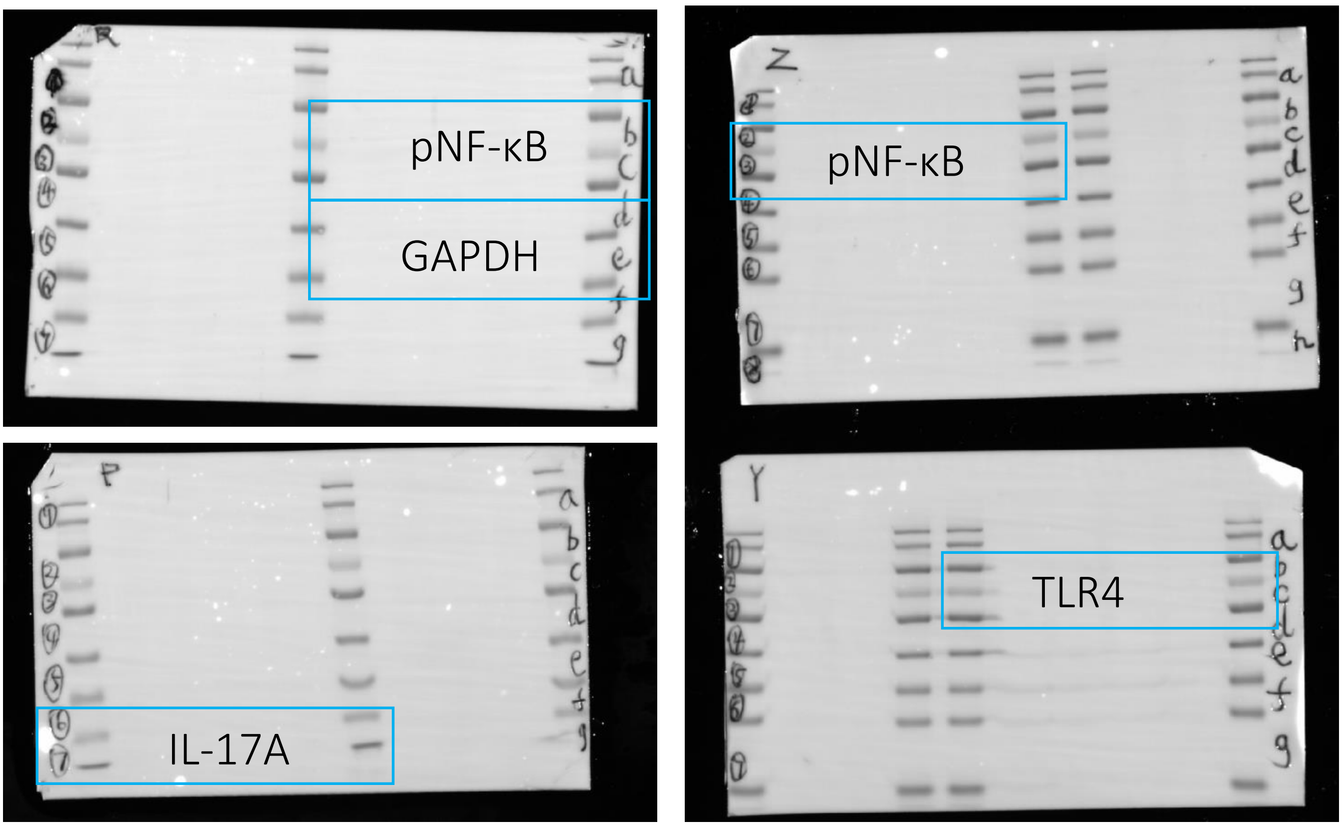

Figure 5

5E

COX-2

ATF4

IL-17A

GAPDH

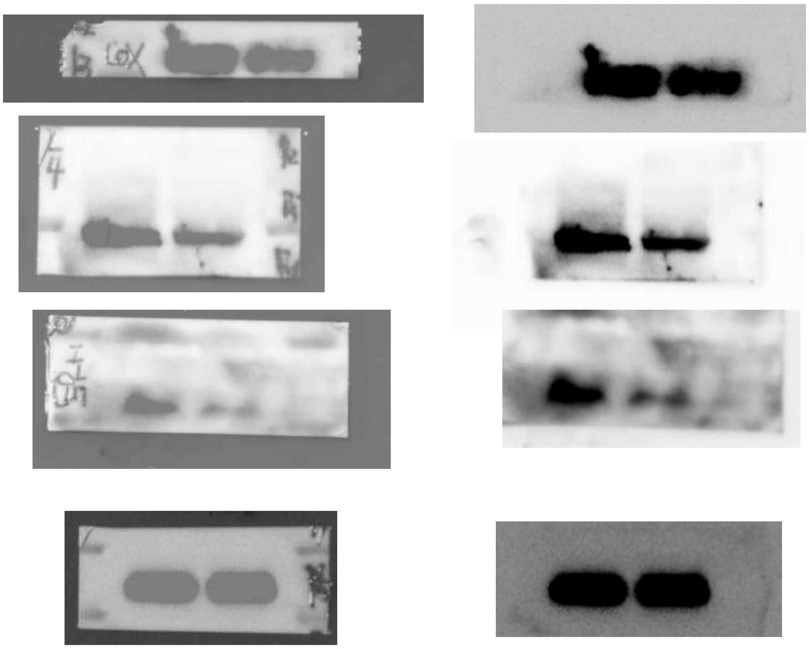

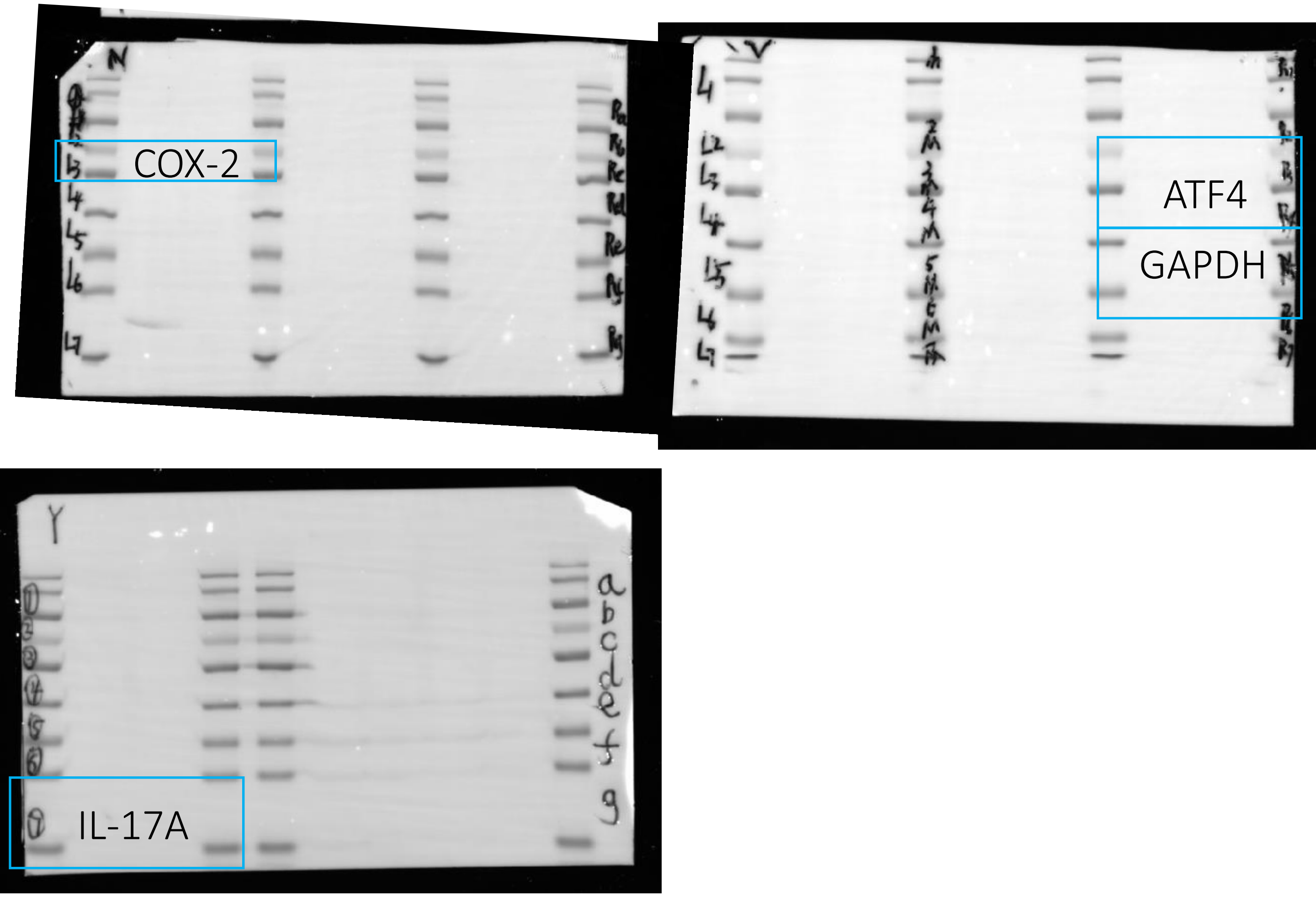

Figure 5

5F

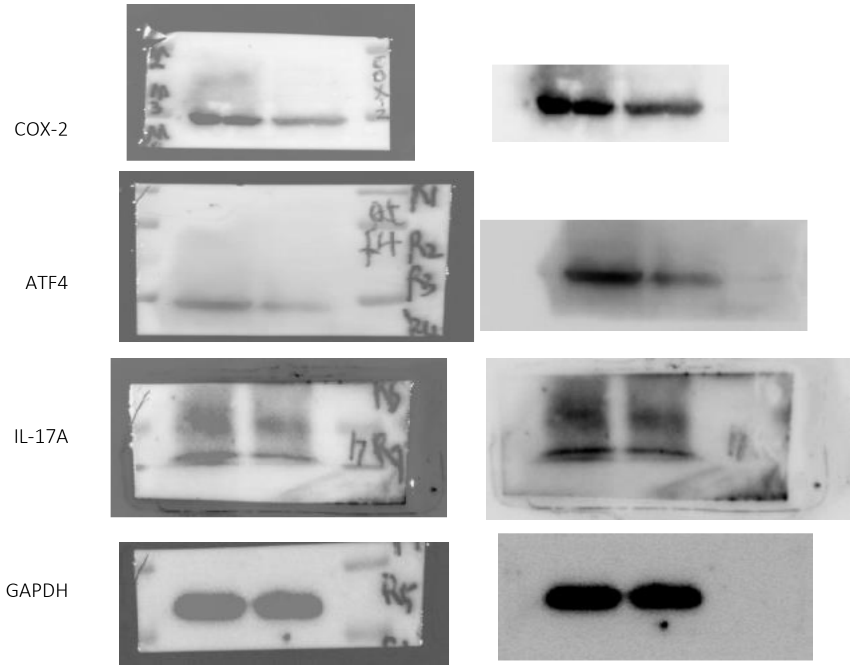

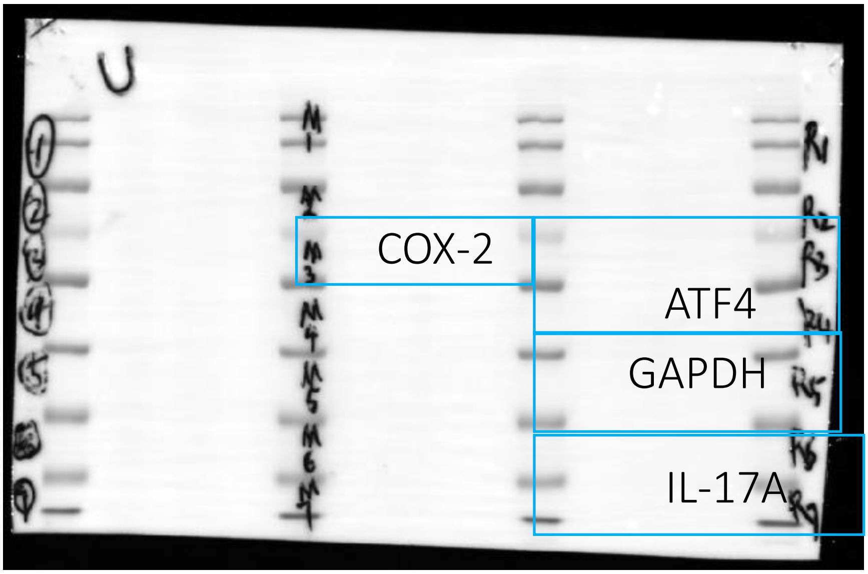

Figure 5

5G

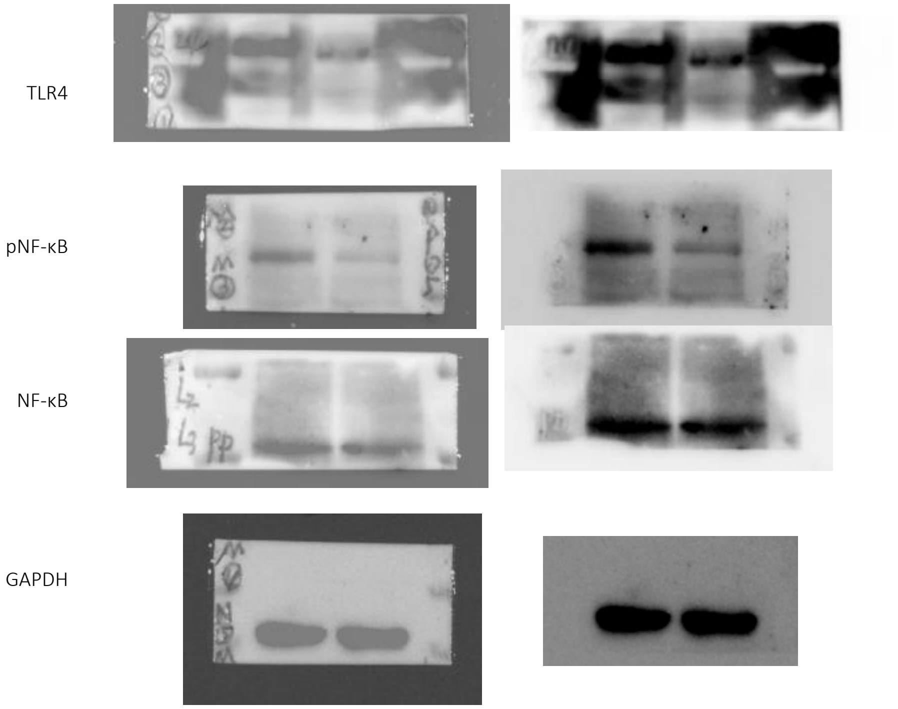

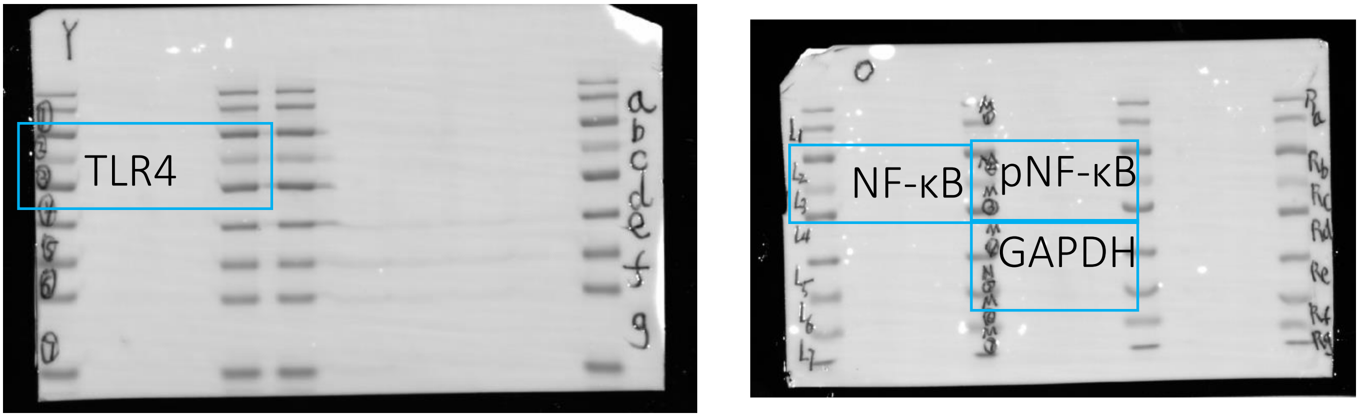

Figure 5

5I

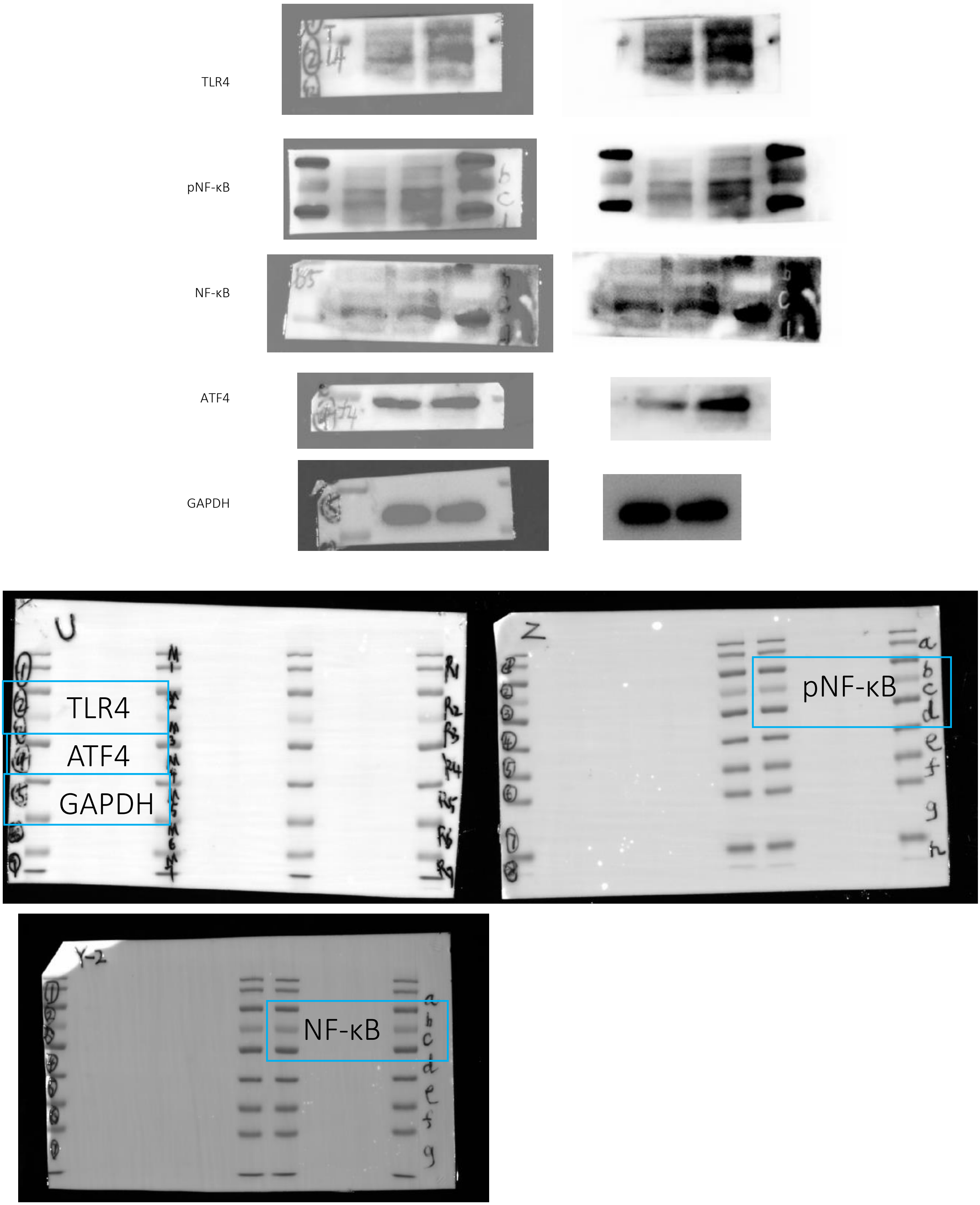

Figure 6

6B

iNOS

Arg1

Iba1

Tubulin

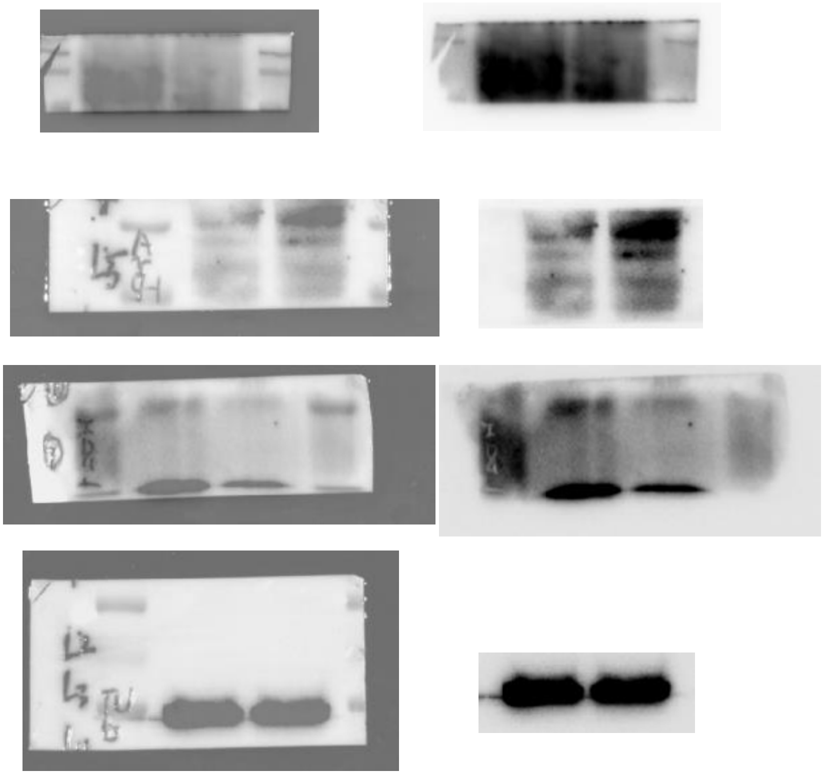

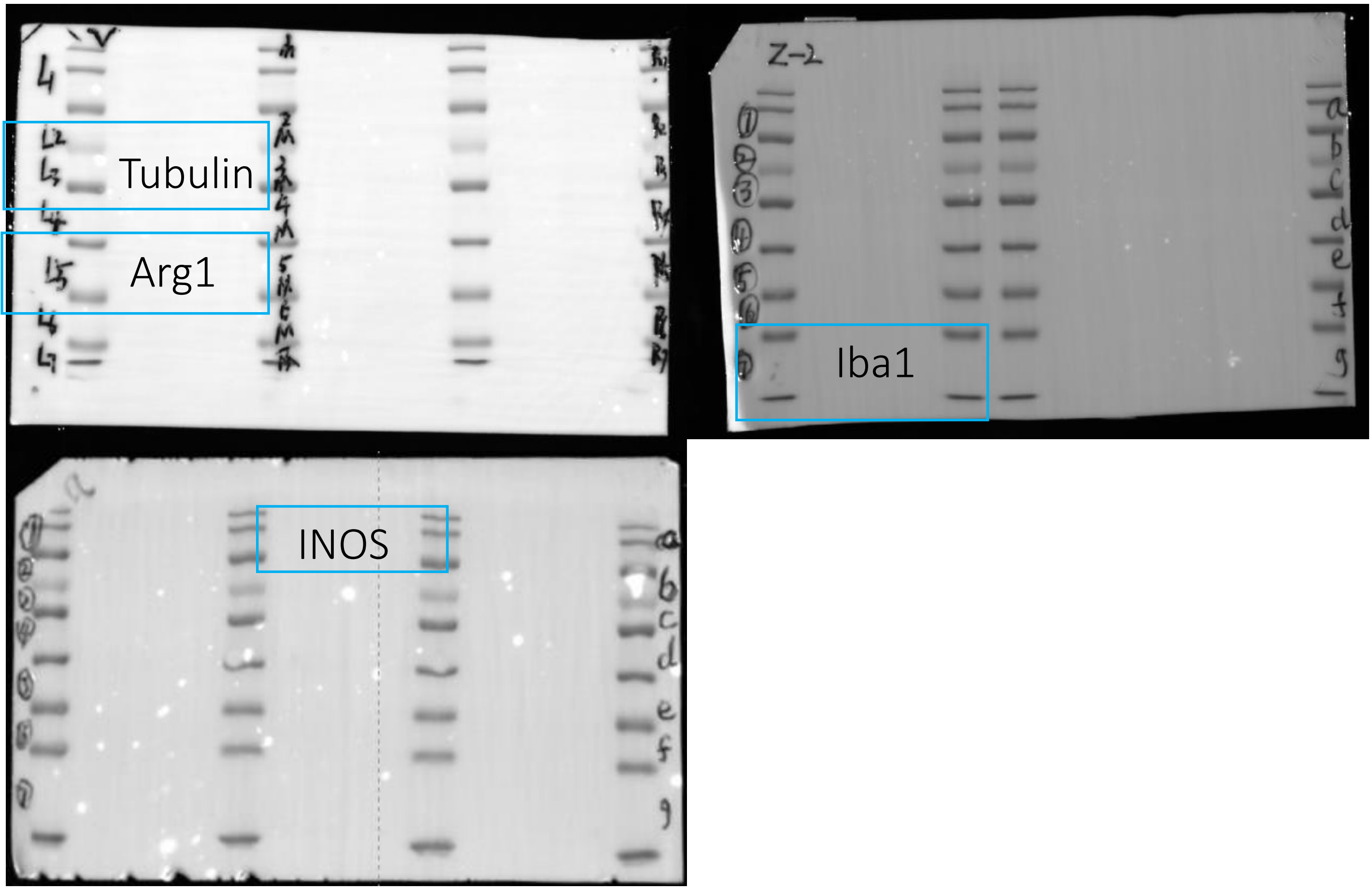

Figure8

8B

TH

GAPDH

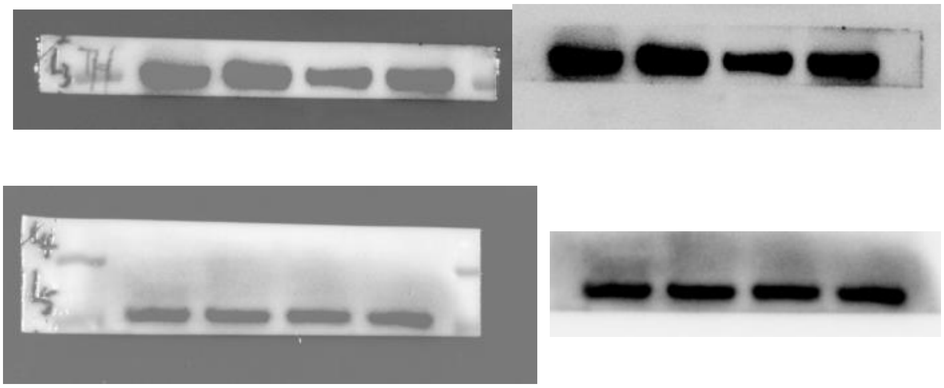

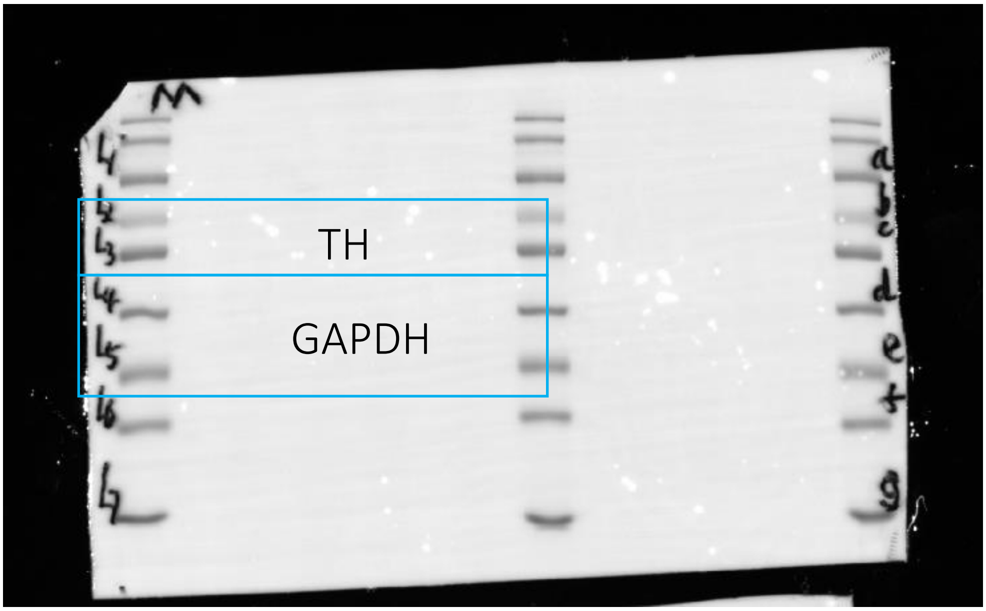

Figure8

8F

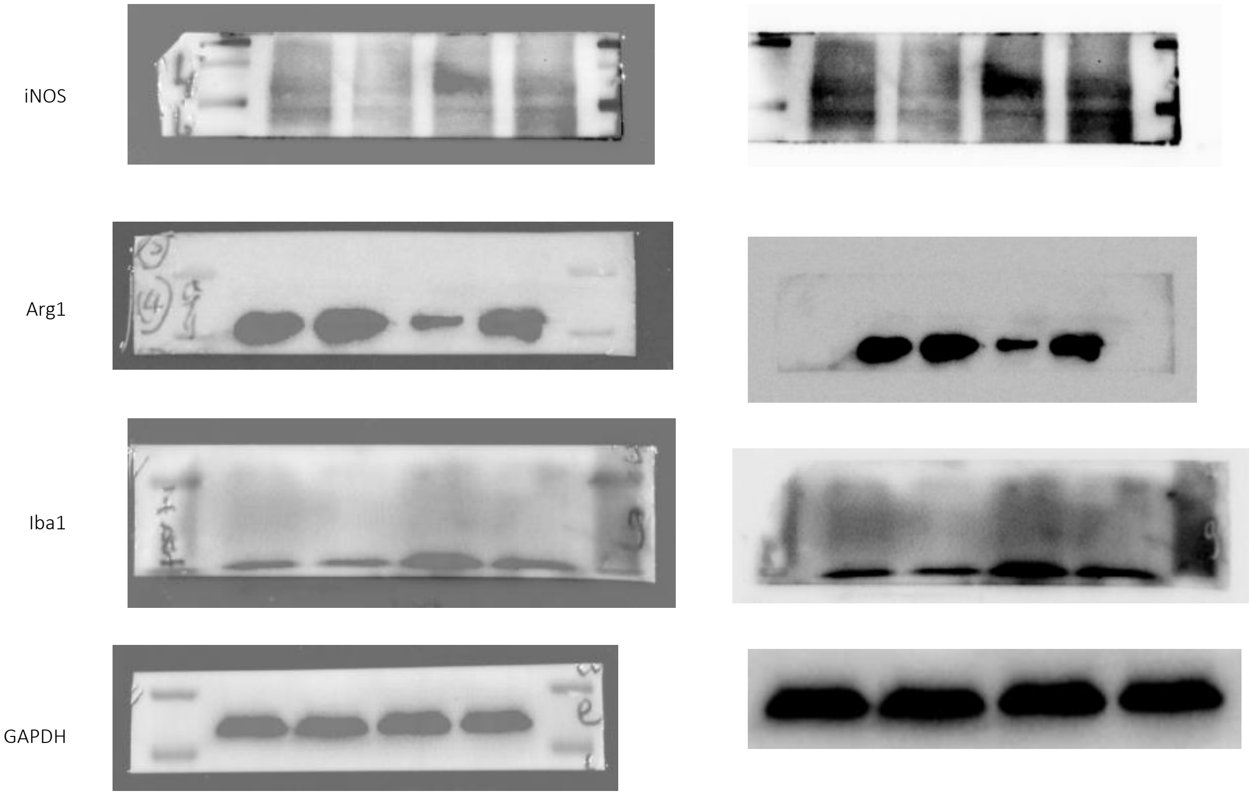

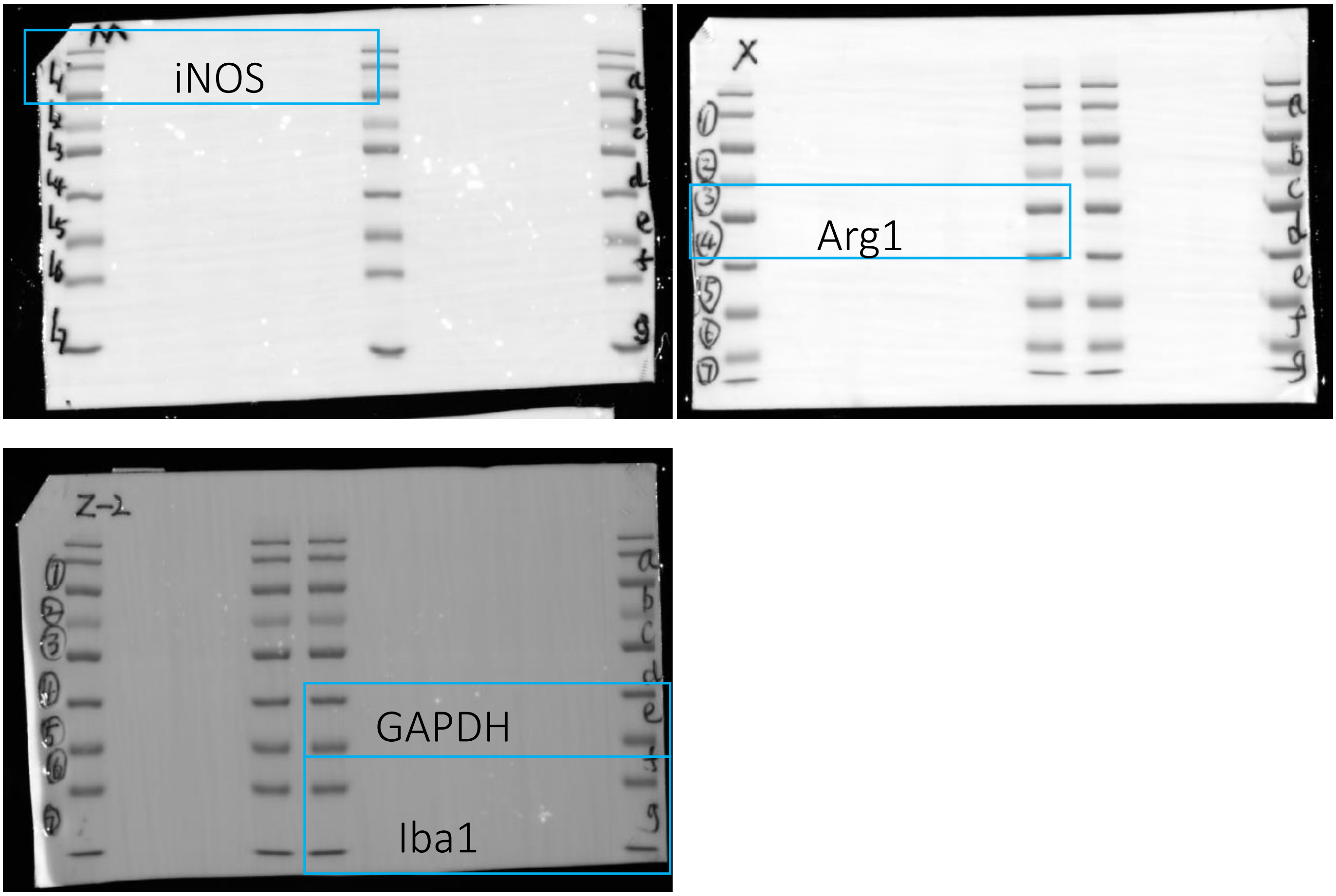

Figure8

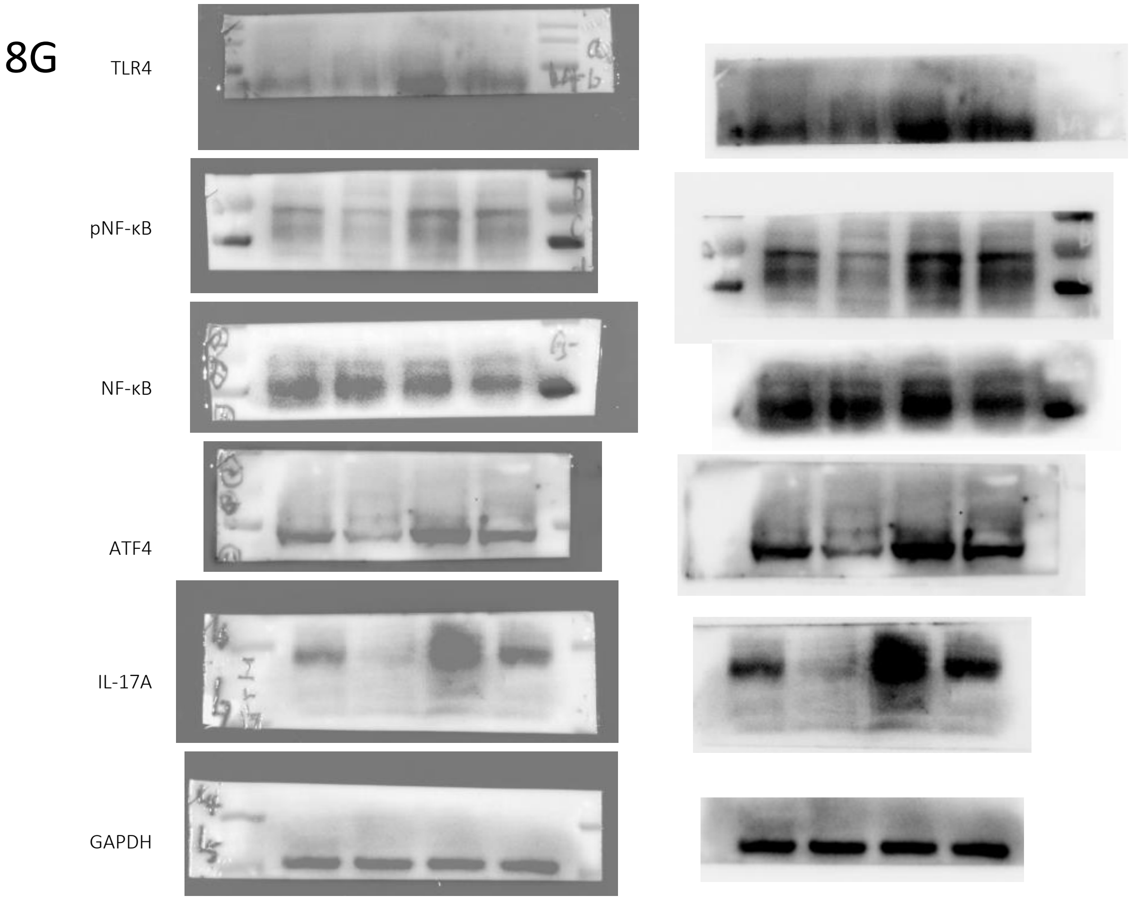
8G
TLR4
pNF-κB
NF-κB
ATF4
IL-17A
GAPDH

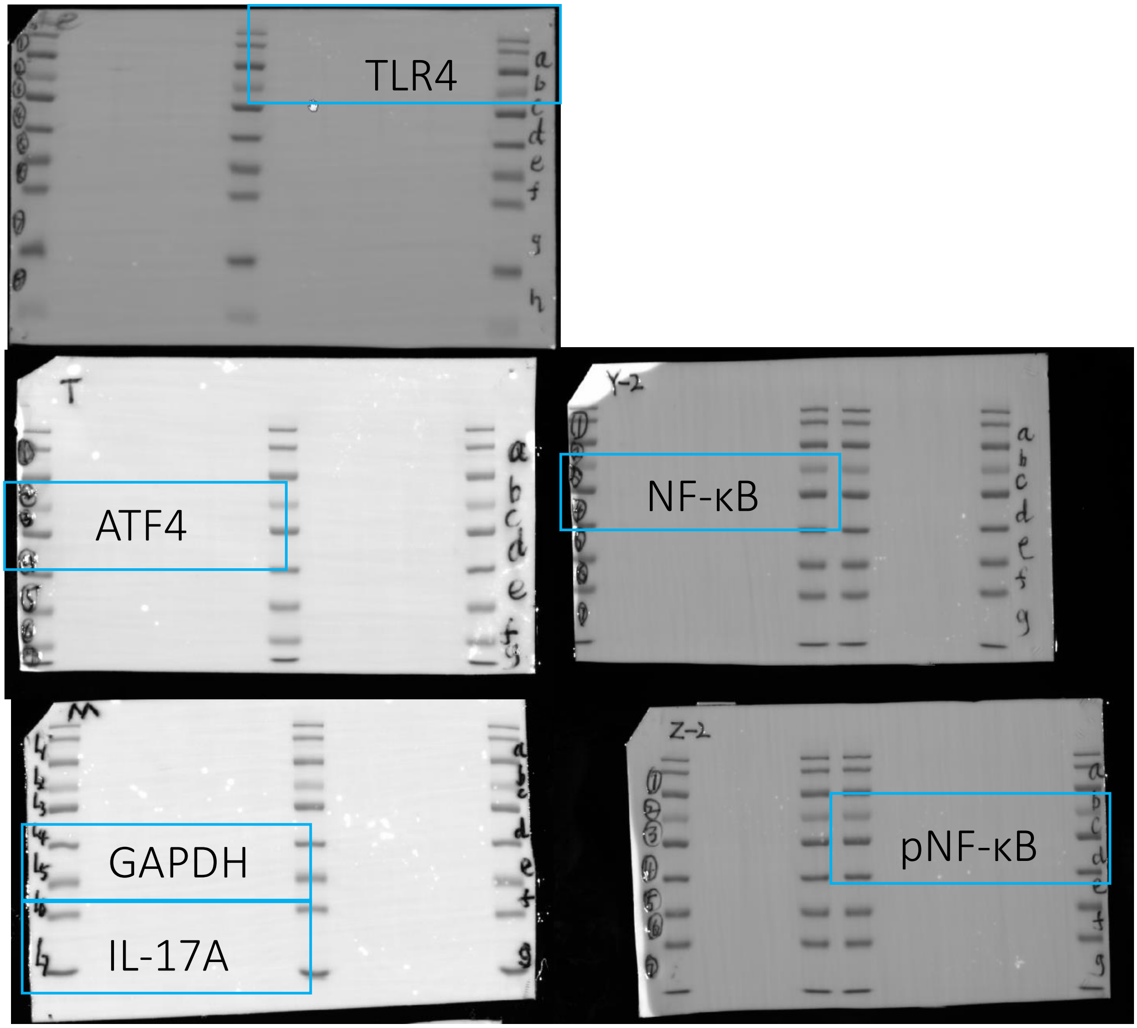
TLR4
ATF4
NF-κB
GAPDH
IL-17A
pNF-κB
